# Targeting Daily Positive Events to Improve Emotional and Functional Well-Being in Adults With Fibromyalgia: Insights From the LARKSPUR Randomized Controlled Trial

**DOI:** 10.2196/54678

**Published:** 2024-12-10

**Authors:** Anthony Ong, Kenneth Wilcox, M Carrington Reid, Elaine Wethington, Dakota Cintron, Elizabeth Addington, Selin Goktas, Judith Moskowitz

**Affiliations:** 1 Department of Psychology Cornell University Ithaca, NY United States; 2 Department of Medicine Weill Cornell University NYC, NY United States; 3 Department of Medical Social Sciences Northwestern University Chicago, IL United States

**Keywords:** positive affect, chronic pain, chronic condition, long-term pain, positive psychology, positive events, fibromyalgia, mHealth, app, digital technology, digital interventions, gerontology, geriatrics, older adult, aging, well-being

## Abstract

**Background:**

Fibromyalgia is a chronic pain condition characterized by widespread musculoskeletal pain, fatigue, and cognitive difficulties, affecting individuals across all age groups. Positive affect (PA) interventions have shown promise in enhancing emotional well-being and pain management in patients with diverse chronic pain conditions. However, the efficacy of internet-delivered PA interventions for individuals with fibromyalgia remains understudied.

**Objective:**

This randomized controlled trial investigated the efficacy of a web-based PA regulation intervention—Lessons in Affect Regulation to Keep Stress and Pain Under Control (LARKSPUR)—in enhancing emotional and functional well-being among adults with fibromyalgia syndrome.

**Methods:**

A total of 95 participants with fibromyalgia syndrome aged 50 years and older (89/95, 94% female) were randomized to one of two fully automated conditions: (1) LARKSPUR (n=49) or (2) emotion reporting/attention control (n=46). At the postintervention and 1-month follow-up time points, participants completed 7 consecutive, end-of-day, web-based reports capturing positive events (PE), pain, fatigue, PA, and negative affect.

**Results:**

Compared to control, LARKSPUR resulted in greater improvements in daily affective responsivity to PE at the postintervention time point, including greater reductions in negative affect (*b_L_*–*b_C_*=–0.06, 95% highest posterior density interval [HPD] –0.10 to –0.02) and increases in PA (*b_L_*–*b_C_*=0.10, 95% HPD 0.02-0.19). Furthermore, across the postintervention and 1-month follow-up time points, LARKSPUR led to greater reductions in pain (*b_L_*–*b_C_*=–0.20, 95% HPD –0.36 to –0.04) and fatigue (*b_L_*–*b_C_*=–0.24, 95% HPD –0.41 to –0.06) following PE.

**Conclusions:**

This randomized controlled trial provides initial evidence that a web-based PA skills intervention can enhance emotional well-being and reduce pain and fatigue in aging adults with fibromyalgia.

**Trial Registration:**

ClinicalTrials.gov NCT04869345; https://clinicaltrials.gov/study/NCT04869345

## Introduction

### Background

Chronic pain is a major public health problem affecting millions of people worldwide and imposing significant burdens on individuals’ well-being and quality of life. Among chronic pain conditions, fibromyalgia syndrome (FMS) is one of the most prevalent and challenging to treat. FMS involves widespread musculoskeletal pain, fatigue, and debilitating symptoms that impair physical and psychological functioning [1-3]. While pharmacological treatments provide partial relief for some individuals, nonpharmacologic approaches are often needed to improve well-being [4]. Consequently, innovative interventions are required that address the multifaceted impacts of FMS on daily life.

In recent years, positive psychological interventions have emerged as a promising approach to promote well-being and enhance mental health outcomes [5-7]. Integrating daily positive activities into chronic pain treatment has shown the potential to foster resilience and improve overall well-being [8,9]. Daily positive events (PE)—small, meaningful experiences that increase positive emotions [10,11], such as enjoying a pleasant meal, having a meaningful conversation, or experiencing moments of comfort—may be especially beneficial for individuals with FMS. First, cultivating positive emotions, like joy, gratitude, and contentment, through daily PE could improve emotional well-being, as these tend to be deficient in this population [12,13]. Second, actively seeking and savoring positive experiences may instill a greater sense of control and agency over one’s life, counterbalancing feelings of helplessness and hopelessness common in chronic pain [14,15]. Third, pleasant daily activities could facilitate social connection and support, which are essential given individuals with FMS often experience isolation due to their condition [16,17]. Finally, engagement in pleasant activities and PE may facilitate healthy habits and routines, promoting sustained well-being and improvements in daily functioning [18,19].

Internet-delivered positive psychological interventions represent a promising approach to enhancing well-being among those affected by chronic pain, including those with FMS. Web-based programs can overcome barriers to in-person treatment while providing effective tools to boost positive experiences. One such intervention has been widely tested in multiple studies with more than 1000 participants (aged 16 to 78 years) coping with varied life stressors, from diagnosis with a serious illness to daily stress [20-23]. The program has been implemented in person (individually and in groups) and, most recently, has been delivered digitally as a self-guided program for individuals with diabetes [24], depression [25-27], HIV [28], and cancer [21,29] and for the general public during the COVID-19 pandemic [30]. However, research is lacking on how to optimally design such interventions specifically for aging adults with FMS [31].

To address this gap, this study tested a web-based program that integrates prior theoretical work on positive emotions [32-34], stress and coping [35,36], and PE [37,38]. Specifically, we examined the effects of an internet-delivered positive affect (PA) skills intervention, called Lessons in Affect Regulation to Keep Stress and Pain Under Control (LARKSPUR), on daily PE, affect, pain, and fatigue in adults with FMS. LARKSPUR is a self-guided web-based intervention that consists of 8 skills taught over the course of 5 weeks with opportunities for practice of the skills built into the platform. Our initial pilot study demonstrated LARKSPUR’s feasibility and preliminary efficacy at improving overall affect and reducing pain catastrophizing [39]. However, it did not examine fluctuations in participants’ daily experiences, which are hypothesized to be a key mechanism based on LARKSPUR’s theoretical underpinnings [34,39].

### Aims and Hypotheses

We sought to determine if the LARKSPUR intervention could improve daily emotional and functional well-being in adults with FMS by targeting daily PE. Consistent with the Positive Pathways to Health model [34], we hypothesized that participants randomly assigned to LARKSPUR would show greater enhancements in daily PE-related affective well-being, pain, and fatigue compared to participants in the emotion reporting/attention control condition. By using digital technology, this study provides a scalable and accessible intervention that may help overcome barriers to in-person care for individuals with FMS [40,41].

## Methods

### Study Design and Procedure

#### Overview

The LARKSPUR pilot (trial registration: ClinicalTrials.gov NCT04869345) is described elsewhere [42]. Briefly, we recruited participants who met the following criteria: (1) aged 50 years and older, (2) access to a Wi-Fi internet connection, (3) English literacy via self-reports of fluency and reading and writing comprehension, and (4) diagnosis of FMS based on the American College of Rheumatology Fibromyalgia Symptom Severity Scale [43] or physician confirmation of FMS. Exclusions were (1) moderate or severe cognitive impairment (two or more errors on a 6-item Mini-Mental State Examination) [44], (2) current behavioral treatment for chronic pain, or (3) enrollment in another chronic pain trial. Eligible and consenting participants were randomly assigned to LARKSPUR or the control condition.

Participants were randomly assigned to either the intervention group (LARKSPUR) or the control group (emotion reporting) in a 1:1 ratio, using block sizes of 2, 4, 6, or 8. The random allocation sequence was generated centrally by a computerized program and implemented by study staff who were not involved in data collection. To prevent selection bias, allocation concealment was used, and group assignments were revealed to both participants and designated study staff only after the baseline assessment was completed. While outcome assessors were blinded to group assignments, participants could not be blinded due to the nature of the intervention.

#### LARKSPUR Intervention

Individuals randomized to LARKSPUR received skills training to increase PA. The self-guided web-based intervention instructed individuals in the use of 8 PA skills delivered over 5 weekly learning modules. The eight skills included (1) noticing PE [45,46], (2) savoring PE [47,48], (3) identifying personal strengths [49,50], (4) behavioral activation to set and work toward attainable goals [51,52], (5) mindfulness [53,54], (6) positive reappraisal [35,55], (7) gratitude [56,57], and (8) acts of kindness [58,59]. Each module consisted of a video introduction, web-based exercises, examples, and homework assignments. Participants practiced skills daily and reported experiences on the digital platform.

#### Control Program

Control participants completed daily emotion reports, rating positive and negative emotions over the past 24 hours on a 5-point Likert scale. This control condition was designed to match the LARKSPUR group in terms of web-based contact, attention to emotional states, and study duration, without providing any specific skills or strategies to enhance PA or cope with pain. Previous studies have used similar emotion reporting as an active control condition for positive psychology interventions [34].

Participants in both arms were assessed at the baseline, postintervention, and 1-month follow-up time points. In addition, before the intervention (baseline), after the intervention (postintervention), and at 1-month follow-up, participants completed a 7-day burst of web-based daily assessments of PE, PA and negative affect (NA), pain intensity, and fatigue. Participants received up to US $142 in gift cards for completing the assessments: US $75 for baseline, postintervention, and follow-up assessments; US $42 for the diary assessments (US $2 each for 21 days); and US $25 for postintervention feedback.

### Participants

Participants were recruited from July 2021 to June 2022 through referrals from New York State practicing physicians and posted flyers throughout the New York Presbyterian Health Care System, New York City–based senior centers, community centers, and web-based platforms (eg, Facebook groups). Recruitment links were also posted on clinicaltrials.gov and emailed to potential participants via ResearchMatch, a national health volunteer research registry created by several US-based academic institutions and supported by the US National Institutes of Health.

### Measures

The primary outcomes included affective, pain, and fatigue responsivity to daily PE. PE were assessed with five items asking participants to report whether the following PE had occurred in the past 24 hours: (1) positive interpersonal interaction; (2) positive experience at work, school, or a volunteer position; (3) positive experience at home; (4) network PE (ie, PE experienced by a close friend or relative); and (5) any other PE [10].

PA and NA were assessed using the 20-item modified Differential Emotions Scale, which asked respondents to rate how often they felt 10 positive (eg, amusement, gratitude, and joy) and 10 negative (eg, anger, guilt, and sadness) emotions during the past 24 hours [60]. Items were rated on a 5-point Likert-type scale ranging from 0 (never) to 4 (most of the time).

Pain intensity and fatigue were each measured using a single item with a rating scale from 0 (no pain or fatigue) to 10 (as much pain or fatigue as could be) [61].

### Statistical Analysis

#### Overview

Given the relatively modest sample size and longitudinal data structure, we used Bayesian estimation rather than frequentist estimation. Compared to frequentist methods, Bayesian methods are better powered for modeling complex data with limited observations, as they incorporate both sample evidence and prior knowledge to derive posterior distributions [62]. To enable effect size interpretation, we report Bayesian analogs to frequentist intervals and *P* values: highest posterior density intervals (HPDs) and posterior probabilities of direction (*P_d_*). HPDs describe uncertainty by delineating the most credible values comprising a certain percentage of the posterior distribution [63]. For example, a 95% HPD of 0.1 to 0.3 indicates a 95% probability that the true effect lies between 0.1 and 0.3 based on the accumulated evidence. The *P_d_* value directly quantifies certainty regarding an effect’s existence and direction [64].

We computed *P_d_* using the *bayestestR* (version 0.13.1) R package (R Foundation for Statistical Computing) [65]. Although related to frequentist *P* values, *P_d_* offers advantages in interpreting effects for small samples [66]. If 99% of the posterior distribution lies above zero, there is high certainty of a positive effect. We used a *P_d_* threshold of .975, corresponding to a frequentist significance level of .05 for 2-sided testing: effects with a *P_d_*>.975 were considered statistically “significant.” Overall, this Bayesian framework enabled appropriate hypothesis testing despite the limited sample size.

#### Measurement Error

We accounted for measurement errors in PA and NA using a Bayesian errors-in-variables approach [67-69]. For PA and NA, person-specific measurement error in each 7-day burst was accounted for using Williams and Hazer’s [70] approach, which uses Cronbach α [71] as a lower bound for reliability (average within-person reliabilities for PA and NA were 0.93 and 0.90, respectively). As a sensitivity check, we also conducted analyses without adjusting for measurement error; results were robust to measurement error correction. Measurement error correction was not performed for the single-item pain and fatigue outcomes.

#### Longitudinal Mixed-Effects Models

Outcomes were assessed using intention-to-treat analyses for all participants providing data at baseline and at least 1 postintervention or 1-month follow-up assessment. Data were modeled using multivariate longitudinal mixed-effects models [72] within a Bayesian framework [73] using the *brms* R package (version 2.20.4) [74] with default noninformative priors. Specifically, we modeled PA and NA as bivariate outcomes, where PA was normally distributed and NA was lognormally distributed, to account for notable positive skew potentially arising from censoring [75] (regression coefficients for NA represent the multiplicative change in NA for a one-unit predictor increase); we modeled pain and fatigue as correlated multivariate normally distributed outcomes.

The study included three key time points for data collection: baseline (T1), postintervention (T2), and 1-month follow-up (T3). Baseline measures were collected immediately before the intervention began. The postintervention assessment (T2) was conducted immediately after the 5-week intervention period, allowing us to capture the immediate effects of the LARKSPUR program. The 1-month follow-up (T3) was designed to assess the durability of the intervention effects over time. In our analyses, we used effect coding to represent the treatment group and time points.

For the treatment group, LARKSPUR was coded as 0.5 and the control condition as –0.5. For the time points used in the main analysis, the 1-month follow-up (T3) was coded as 0.5 and the postintervention time point (T2) was coded as –0.5. Treatment group (0.5=LARKSPUR, –0.5=control) and time point (0.5=1-month follow-up, –0.5=postintervention) were effect-coded. We adjusted for grand-mean-centered baseline PE for all outcomes. We also adjusted each outcome for its corresponding grand-mean-centered baseline score (eg, PA was predicted by grand-mean-centered baseline PA, NA was predicted by grand-mean-centered baseline NA, pain was predicted by grand-mean-centered baseline pain, and fatigue was predicted by grand-mean-centered baseline fatigue). The linear daily change was assumed for all outcomes and centered at the middle (fourth day) of a 7-day burst.

Between-person and within-person effects of PE were disaggregated by including individual burst-specific average PE (grand-mean-centered) and person-mean-centered daily PE as predictors of all outcomes [76]. Finally, we assessed the moderation effects of within-person and between-person PE on treatment differences and treatment differences in change by including 3-way interactions (average PE×group×time and daily PE×group×time) and lower-order, 2-way interactions among treatment group, time, and daily or average PE. Planned comparisons examined between-group differences in PE responsivity at the postintervention and follow-up time points.

### Ethical Considerations

Ethics approval was obtained from the Institutional Review Board at Weill Cornell Medicine (protocol number 20-06022291). All participants were informed about the study details and procedures before providing their written consent. The collected data were coded and deidentified. Participation was strictly voluntary, and participants were informed about their right to withdraw from the study at any point without any consequences.

## Results

### Demographic Characteristics

**Figure 1:**
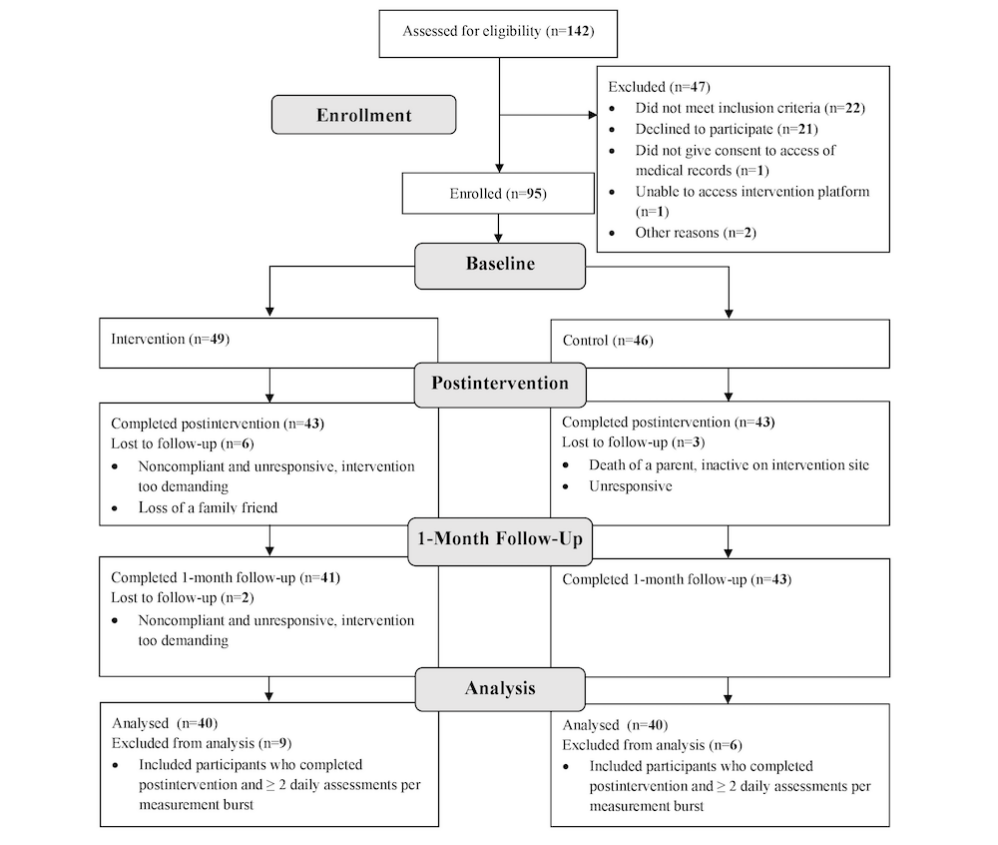
CONSORT (Consolidated Standards of Reporting Trials) flow diagram for the LARKSPUR study. LARKSPUR: Lessons in Affect Regulation to Keep Stress and Pain Under Control.

The sample included 95 adults aged 50-80 years. The majority were female (89/95, 94%) and identified as White (76/95, 80%). Of the 142 individuals initially screened for participation, 47 individuals were excluded from the study. The primary reasons for exclusion were not meeting the inclusion criteria (n=22) and declining to participate (n=21). Additional reasons included not providing consent for access to medical records (n=1), inability to access the intervention platform (n=1), and other unspecified reasons (n=2). The remaining 95 eligible participants were enrolled in the study and randomly assigned to either the LARKSPUR intervention or the control condition. All 95 participants completed the baseline questionnaires and were randomly assigned to the LARKSPUR intervention (n=49) or the control (n=46). Using an intention-to-treat analysis, 86 participants (n=43 LARKSPUR and n=43 control) completing baseline and postintervention assessments were included. In addition, participants completing ≥2 daily assessments per measurement burst were included to assess daily changes, resulting in a final analytic sample of 80 participants (n=40 in LARKSPUR and n=40 in control; see the CONSORT [Consolidated Standards of Reporting Trials] diagram in Figure 1). Demographic variables and baseline measures for the analytic sample are summarized in Table 1. As demographic and baseline measures were collected prerandomization, group differences at baseline were not tested [77].

**Table 1:** Demographic characteristics and baseline measures of participants in LARKSPUR^a^ and the control condition.

Characteristic			LARKSPUR (n=40)		Control (n=40)		Overall (n=80)
**Sex, n (%)**							
	Male	1 (2)		3 (8)		4 (5)	
	Female	39 (98)		37 (92)		76 (95)	
**Age group (years), n (%)**							
	50-59	17 (42)		21 (52)		38 (48)	
	60-69	16 (40)		17 (42)		33 (41)	
	70-79	6 (15)		2 (5)		8 (10)	
	≥80	1 (2)		0 (0)		1 (1)	
**Race and ethnicity, n (%)**							
	Hispanic or Latino	1 (2)		2 (5)		3 (4)	
	Black or African American	2 (5)		4 (10)		6 (8)	
	White	33 (82)		32 (80)		65 (81)	
	More than one race	3 (8)		1 (2)		4 (5)	
	Not reported	1 (2)		1 (2)		3 (4)	
**Education, n (%)**							
	High school diploma or GED^b^	2 (5)		1 (2)		3 (3)	
	Some college (no degree)	9 (22)		8 (20)		17 (20)	
	Associate degree	6 (15)		4 (10)		11 (13)	
	Bachelor’s degree	10 (25)		8 (20)		21 (24)	
	Postgraduate (no degree)	3 (8)		7 (18)		10 (12)	
	Master’s degree	8 (20)		12 (30)		21 (24)	
	Doctoral degree	2 (5)		0 (0)		3 (3)	
**Baseline measure, mean (SD)**							
	Positive events	2.2 (0.9)		2.3 (1.0)		2.2 (0.9)	
	Positive affect	2.7 (0.6)		2.7 (0.6)		2.7 (0.6)	
	Negative affect	1.7 (0.5)		1.5 (0.5)		1.6 (0.5)	
	Pain	5.5 (1.5)		5.5 (1.8)		5.5 (1.7)	
	Fatigue	6.0 (1.7)		5.4 (2.1)		5.7 (1.9)	

^a^LARKSPUR: Lessons in Affect Regulation to Keep Stress and Pain Under Control.

^b^GED: General Education Degree.

### Main Analysis

#### Affective Responsivity

**Figure 2:**
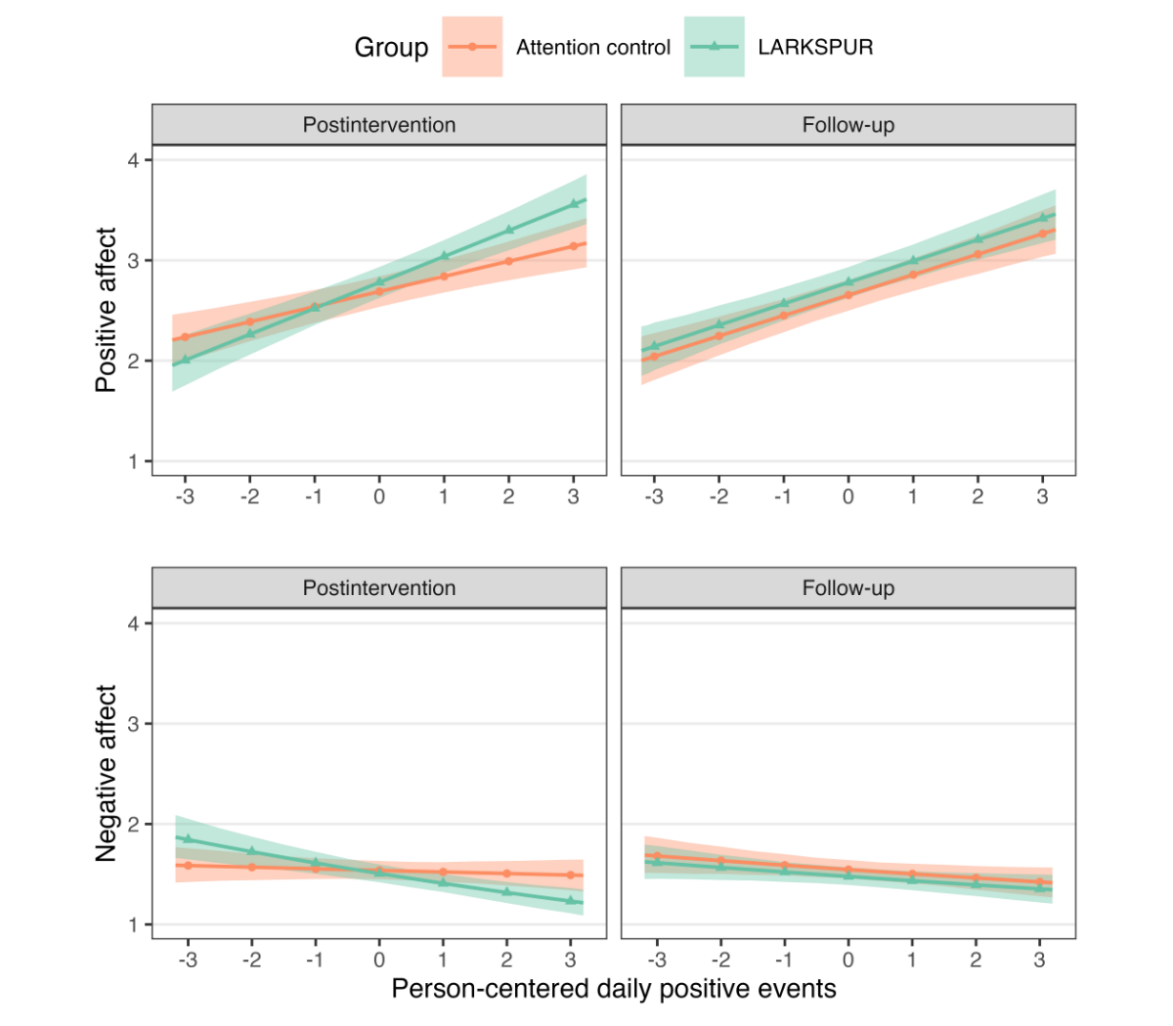
Predicted responsivity to daily positive events from mixed-effects model for positive affect and negative affect at the postintervention and 1-month follow-up time points. LARKSPUR: Lessons in Affect Regulation to Keep Stress and Pain Under Control.

The bivariate mixed-effects model revealed significant interactions between treatment group, time point, and daily PE in predicting PA and NA. Parameter posterior median estimates and 95% HPD intervals are presented in Table 2. In Bayesian estimation, the distribution of model parameters (eg, regression slopes) is estimated, allowing for a comprehensive characterization of uncertainty. To summarize the typical estimate or central tendency of these distributions, we report median parameter estimates. These estimates can be interpreted in a manner analogous to traditional frequentist estimates obtained through maximum likelihood or least-squares estimation, representing the most probable value of a parameter given the accumulated evidence. Furthermore, 95% HPD intervals provide a quantitative measure of uncertainty in parameter estimates, analogous to frequentist CIs. These intervals can be directly interpreted as follows: given the model and data, there is a 95% probability that the true parameter value lies within the HPD interval, thereby providing a range of plausible values for the parameter. Interactions indicate that LARKSPUR differentially impacted affective responsivity to daily PE compared to the control condition. Specifically, planned comparisons (ie, differences) between regression slopes for LARKSPUR (*b_L_*) and controls (*b_C_*), denoted by *b_L_*–*b_C_*, showed that at the postintervention time point, LARKSPUR participants exhibited greater reductions in NA (*b_L_*–*b_C_*=–0.06, 95% HPD –0.10 to –0.02; *P_d_*>.99) and increases in PA (*b_L_*–*b_C_*=0.10, 95% HPD 0.02-0.19, *P_d_*=.99) in response to daily PE compared to controls. However, these differential gains in affective responsivity were not maintained at 1-month follow-up, with nonsignificant between-group differences in both PA (*b_L_–b_C_*=0.01, 95% HPD –0.08 to 0.09; *P_d_*=.56) and NA (*b_L_*–*b_C_*=–0.00, 95% HPD –0.04 to 0.04; *P_d_*=.51) responsivity. As shown in Figure 2, both groups evidenced slightly diminished affective responsivity from postintervention to follow-up, suggesting that continued practice of LARKSPUR skills may be necessary to sustain affective gains long-term.

**Table 2:** Mixed-effects model parameter estimates for PA^a^ and NA^b^ (n=80).

			PA, coefficient (95% HPD^c^)		NA, coefficient (95% HPD)
**Fixed effects**					
	Intercept	2.72 (2.62 to 2.83)		0.41 (0.37 to 0.45)	
	Day	–0.03 (–0.04 to –0.01)		–0.01 (–0.01 to –0.00)	
	Baseline outcome	0.75 (0.58 to 0.92)		0.47 (0.40 to 0.55)	
	Time	–0.02 (–0.09 to 0.05)		–0.01 (–0.03 to 0.02)	
	Group	0.11 (–0.10 to 0.32)		–0.03 (–0.11 to 0.05)	
	PE^d^ (BW^e^)	0.17 (0.06 to 0.27)		–0.01 (–0.05 to 0.03)	
	PE (WI^f^)	0.21 (0.17 to 0.24)		–0.04 (–0.05 to –0.02)	
	Time×group	0.02 (–0.11 to 0.16)		–0.02 (–0.07 to 0.03)	
	Group×PE (BW)	0.06 (–0.13 to 0.25)		0.01 (–0.06 to 0.09)	
	Time×PE (BW)	0.04 (–0.03 to 0.10)		–0.02 (–0.04 to 0.01)	
	Group×PE (WI)	0.06 (–0.01 to 0.13)		–0.03 (–0.06 to 0.00)	
	Time×PE (WI)	0.00 (–0.05 to 0.05)		0.01 (–0.01 to 0.04)	
	Time×group×PE (BW)	–0.03 (–0.16 to 0.10)		–0.02 (–0.07 to 0.03)	
	Time×group×PE (WI)	–0.10 (–0.20 to –0.00)		0.06 (0.01 to 0.11)	
**Variance parameters**					
	L1^g^ SD	0.34 (0.31 to 0.36)		0.15 (0.14 to 0.16)	
	L1 AR(1)^h^	0.42 (0.30 to 0.53)		0^i^	
	L1 *r*	0		0	
	L2^j^ SD: intercepts	0.43 (0.36 to 0.53)		0.17 (0.14 to 0.20)	
	L2 SD: PE (WI) slopes	0.10 (0.07 to 0.15)		0.04 (0.03 to 0.06)	
	L2 *r*: intercepts	–0.30 (–0.52 to –0.05)		0	
	L2 *r*: PE (WI) slopes	–0.78 (–0.97 to –0.38)		0	

^a^PA: positive affect.

^b^NA: negative affect.

^c^HPD: highest posterior density.

^d^PE: positive events.

^e^BW: between-person effect.

^f^WI: average within-person effect.

^g^L1: level 1 (within person).

^h^AR(1): lag-1 residual autocorrelation.

^i^Not estimated.

^j^L2: level 2 (between person).

#### Pain and Fatigue Responsivity

**Figure 3:**
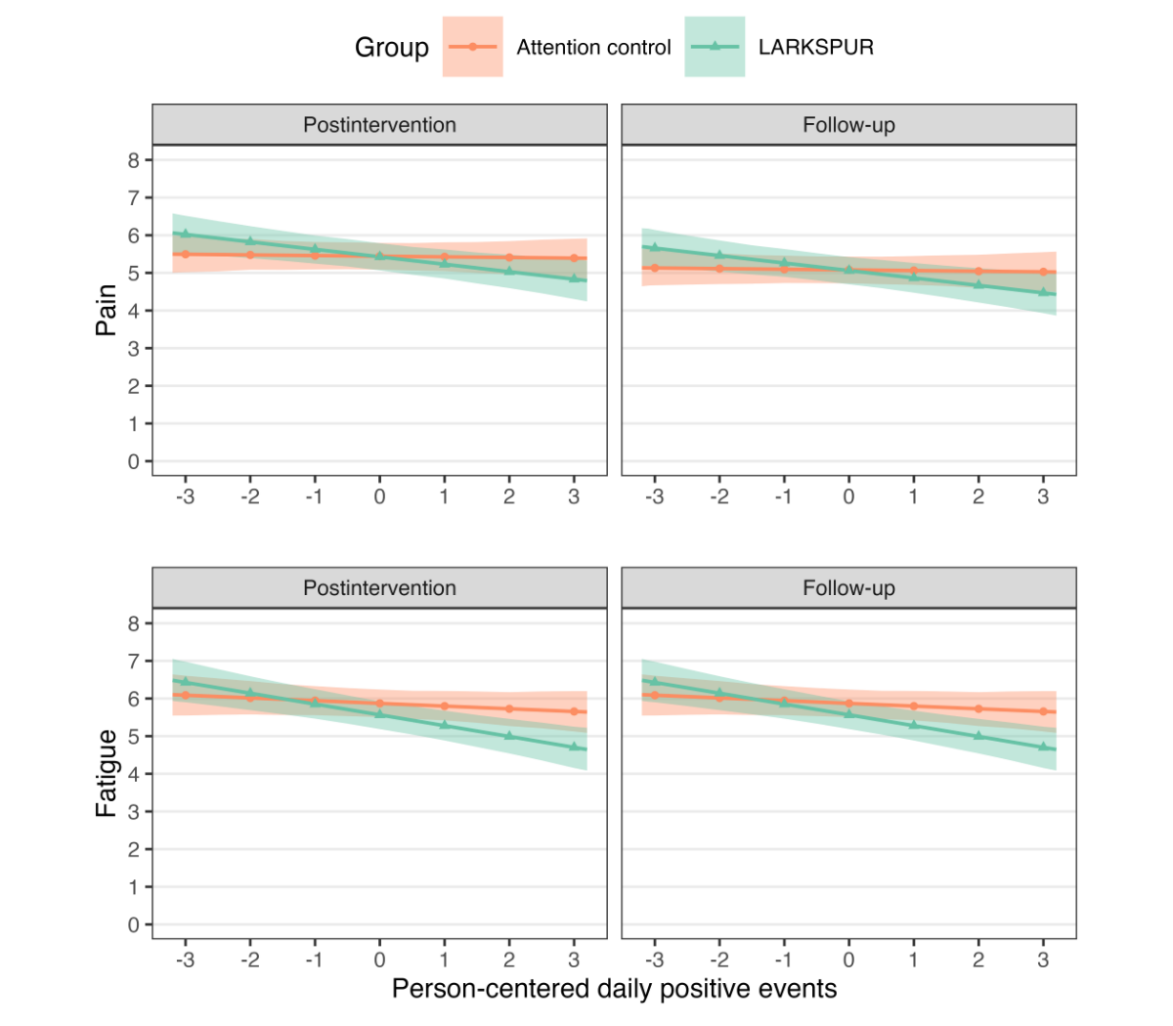
Predicted responsivity to daily positive events from mixed-effects model for pain and fatigue at the postintervention and 1-month follow-up time points. LARKSPUR: Lessons in Affect Regulation to Keep Stress and Pain Under Control.

In contrast to the affective outcomes, LARKSPUR yielded more persistent improvements in daily pain and fatigue responsivity compared to the control condition (Table 3). Across the postintervention and 1-month follow-up time points, LARKSPUR led to greater reductions in pain (*b_L_*–*b_C_*=–0.20, 95% HPD –0.36 to –0.04; *P_d_*=.99) and fatigue (*b_L_*–*b_C_*=–0.24, 95% HPD –0.41 to –0.06; *P_d_*>.99) following PE. As illustrated in Figure 3, LARKSPUR participants maintained diminished pain and fatigue following daily PE from the postintervention time point through 1-month follow-up, whereas controls showed minimal change. This sustained effect for the functional outcomes indicates that LARKSPUR may have produced broader impacts beyond temporary affective gains. In sum, results suggest LARKSPUR (relative to the control condition) improved affective, pain, and fatigue responsivity to daily PE, particularly at the postintervention time point. The affective benefits were not maintained, but the pain and fatigue improvements persisted through the 1-month follow-up.

**Table 3:** Mixed-effects model parameter estimates for pain and fatigue (n=80).

			Pain, coefficient (95% HPD^a^)		Fatigue, coefficient (95% HPD)
**Fixed effects**					
	Intercept	5.24 (4.99 to 5.49)		5.75 (5.48 to 6.02)	
	Day	–0.01 (–0.06 to 0.03)		–0.04 (–0.09 to 0.00)	
	Baseline outcome	0.79 (0.64 to 0.94)		0.76 (0.62 to 0.91)	
	Time	–0.37 (–0.56 to –0.18)		–0.06 (–0.26 to 0.14)	
	Group	–0.02 (–0.52 to 0.49)		–0.34 (–0.89 to 0.21)	
	PE^b^ (BW^c^)	–0.05 (–0.28 to 0.20)		0.00 (–0.26 to 0.26)	
	PE (WI^d^)	–0.12 (–0.20 to –0.04)		–0.20 (–0.28 to –0.11)	
	Time×group	–0.33 (–0.72 to 0.05)		–0.33 (–0.74 to 0.07)	
	Group×PE (BW)	0.35 (–0.13 to 0.83)		0.07 (–0.44 to 0.59)	
	Time×PE (BW)	0.03 (–0.15 to 0.21)		0.11 (–0.08 to 0.30)	
	Group×PE (WI)	–0.20 (–0.36 to –0.04)		–0.24 (–0.42 to –0.07)	
	Time×PE (WI)	0.05 (–0.11 to 0.20)		0.09 (–0.09 to 0.26)	
	Time×group×PE (BW)	–0.25 (–0.61 to 0.12)		–0.30 (–0.68 to 0.09)	
	Time×group×PE (WI)	0.10 (–0.21 to 0.42)		–0.02 (–0.37 to 0.33)	
**Variance parameters**					
	L1^e^ SD	1.32 (1.26 to 1.38)		1.45 (1.38 to 1.52)	
	L1 AR(1)^f^	0.19 (0.12 to 0.26)		0.13 (0.06 to 0.20)	
	L1 *r*	0.38 (0.33 to 0.44)		0	
	L2^g^ SD: intercepts	1.03 (0.84 to 1.25)		1.11 (0.92 to 1.35)	
	L2 SD: PE (WI) slopes	0.08 (0.00 to 0.19)		0.08 (0.00 to 0.23)	
	L2 *r*: intercepts	0.37 (0.11 to 0.58)		0	
	L2 *r*: PE (WI) slopes	0.14 (–0.76 to 0.85)		0	

^a^HPD: highest posterior density interval.

^b^PE: positive events.

^c^BW: between-person effect.

^d^WI: average within-person effect.

^e^L1: level 1 (within person).

^f^AR(1): lag-1 residual autocorrelation.

^g^L2: level 2 (between person).

## Discussion

### Principal Findings

This randomized controlled trial provides initial evidence that LARKSPUR, an internet-delivered PA skills intervention, can enhance responsivity to daily PE in adults with FMS. LARKSPUR led to greater decreases in NA and increases in PA following PE compared to the control condition. It also led to reductions in pain and fatigue following PE at the postintervention and 1-month follow-up time points. These findings suggest that LARKSPUR has the potential to be an accessible and effective eHealth intervention to boost well-being and improve symptom management in this population.

### Strengths and Implications

The findings have several strengths and implications for research and practice. First, the results support previous research showing the benefits of positive psychological interventions for populations with chronic pain [13,78]. By targeting engagement in pleasant activities and PE, LARKSPUR aligns with prior work highlighting the value of positive experiences in promoting resilience and well-being [40,41]. Second, the study demonstrates the feasibility and potential of delivering such programs remotely to aging adults with FMS. The web-based format helps overcome barriers to in-person treatment while improving reach, flexibility, and scalability [79-81]. This is especially relevant given COVID-19 contexts demanding flexible health services [82]. Third, incorporating technology into the intervention likely enhanced program quality and engagement through personalized feedback and reminders. These digital components may have facilitated the gains in PE responsivity observed in the LARKSPUR group compared to controls. Fourth, the adaptable nature of LARKSPUR allows for implementation across varied formats to meet diverse user needs and preferences. This tailoring can boost engagement, which is key for patients with chronic pain who have heterogeneous motivations and capacities [8,13]. Finally, the findings provide insights into mechanisms of change. The affective benefits at the postintervention time point suggest LARKSPUR honed positive emotion regulation skills; however, continued practice may be needed to maintain gains. In contrast, the persistence of LARKSPUR’s effects on pain and fatigue through 1-month follow-up indicates the intervention likely produced broader impacts beyond the temporary enhancement of positive emotions.

### Limitations and Future Directions

Several limitations of this study should be acknowledged. The modest sample size limits the precision and generalizability of our findings, highlighting the need for larger, more diverse samples of individuals with fibromyalgia. Future studies should aim to recruit participants from a broader range of demographic groups to examine the effects of LARKSPUR across different populations. Additionally, our reliance on self-report measures may introduce bias, and incorporating objective assessments, such as clinician observations or actigraphy, could provide more robust evidence. Furthermore, our focus on PE frequency was narrow, and exploring the variety of daily experiences may reveal new avenues for improving overall well-being [83-85]. Finally, longer-term follow-up is needed to determine the sustainability of benefits and to investigate strategies for maintaining gains, such as periodic booster sessions.

### Conclusions

This randomized trial provides preliminary evidence that a web-based PA skills intervention can enhance responsivity to daily PE across emotional and functional domains in adults with FMS. Specifically, the findings indicate LARKSPUR improved participants’ affective, pain, and fatigue responses following daily positive experiences compared to controls. This provides initial support for the efficacy of technology-based platforms, like LARKSPUR, to boost well-being in this population by targeting sensitivity to daily PE. Further research is needed to replicate these results in a larger sample and address optimizing technology to enhance LARKSPUR’s scalability and efficacy for diverse aging adults with fibromyalgia.

## Appendix

Multimedia Appendix 1 CONSORT-eHEALTH checklist (V 1.6.1).

## Abbreviations

CONSORT: Consolidated Standards of Reporting Trials
FMS: fibromyalgia syndrome
HPD: highest posterior density
LARKSPUR: Lessons in Affect Regulation to Keep Stress and Pain Under Control
NA: negative affect
PA: positive affect
PE: positive events

## References

[1] Häuser W, Wolfe F (2012). Diagnosis and diagnostic tests for fibromyalgia (syndrome). Reumatismo.

[2] Sarzi-Puttini P, Giorgi V, Marotto D, Atzeni F (2020). Fibromyalgia: an update on clinical characteristics, aetiopathogenesis and treatment. Nat Rev Rheumatol.

[3] Wolfe F, Clauw DJ, Fitzcharles MA, Goldenberg DL, Katz RS, Mease P, Russell AS, Russell IJ, Winfield JB, Yunus MB (2010). The American College of Rheumatology preliminary diagnostic criteria for fibromyalgia and measurement of symptom severity. Arthritis Care Res (Hoboken).

[4] Flynn DM (2020). Chronic musculoskeletal pain: nonpharmacologic, noninvasive treatments. Am Fam Physician.

[5] Carr A, Cullen K, Keeney C, Canning C, Mooney O, Chinseallaigh E, O’Dowd A (2020). Effectiveness of positive psychology interventions: a systematic review and meta-analysis. J Posit Psychol.

[6] Hendriks T, Schotanus-Dijkstra M, Hassankhan A, de Jong J, Bohlmeijer E (2019). The efficacy of multi-component positive psychology interventions: a systematic review and meta-analysis of randomized controlled trials. J Happiness Stud.

[7] Sin NL, Lyubomirsky S (2009). Enhancing well-being and alleviating depressive symptoms with positive psychology interventions: a practice-friendly meta-analysis. J Clin Psychol.

[8] Braunwalder C, Müller R, Glisic M, Fekete C (2022). Are positive psychology interventions efficacious in chronic pain treatment? A systematic review and meta-analysis of randomized controlled trials. Pain Med.

[9] Iddon JE, Dickson JM, Unwin J (2016). Positive psychological interventions and chronic non-cancer pain: a systematic review of the literature. Int J Appl Posit Psychol.

[10] Sin NL, Ong AD, Stawski RS, Almeida DM (2017). Daily positive events and diurnal cortisol rhythms: examination of between-person differences and within-person variation. Psychoneuroendocrinology.

[11] Zautra AJ, Guarnaccia CA, Dohrenwend BP (1986). Measuring small life events. Am J Community Psychol.

[12] Finan PH, Zautra AJ, Davis MC (2009). Daily affect relations in fibromyalgia patients reveal positive affective disturbance. Psychosom Med.

[13] Finan PH, Garland EL (2015). The role of positive affect in pain and its treatment. Clin J Pain.

[14] Ehde DM, Dillworth TM, Turner JA (2014). Cognitive-behavioral therapy for individuals with chronic pain: efficacy, innovations, and directions for research. Am Psychol.

[15] Müller R, Gertz KJ, Molton IR, Terrill AL, Bombardier CH, Ehde DM, Jensen MP (2016). Effects of a tailored positive psychology intervention on well-being and pain in individuals with chronic pain and a physical disability: a feasibility trial. Clin J Pain.

[16] Wolf LD, Davis MC, Yeung EW, Tennen HA (2015). The within-day relation between lonely episodes and subsequent clinical pain in individuals with fibromyalgia: mediating role of pain cognitions. J Psychosom Res.

[17] Wolf LD, Davis MC (2014). Loneliness, daily pain, and perceptions of interpersonal events in adults with fibromyalgia. Health Psychol.

[18] Wood W, Mazar A, Neal DT (2022). Habits and goals in human behavior: separate but interacting systems. Perspect Psychol Sci.

[19] Wood W, Neal DT (2007). A new look at habits and the habit-goal interface. Psychol Rev.

[20] Carrico AW, Moskowitz JT (2014). Positive affect promotes engagement in care after HIV diagnosis. Health Psychol.

[21] Cheung EO, Cohn MA, Dunn LB, Melisko ME, Morgan S, Penedo FJ, Salsman JM, Shumay DM, Moskowitz JT (2017). A randomized pilot trial of a positive affect skill intervention (lessons in linking affect and coping) for women with metastatic breast cancer. Psychooncology.

[22] Moskowitz JT, Hult JR, Duncan LG, Cohn MA, Maurer S, Bussolari C, Acree M (2012). A positive affect intervention for people experiencing health-related stress: development and non-randomized pilot test. J Health Psychol.

[23] Moskowitz JT, Carrico AW, Cohn MA, Duncan LG, Bussolari C, Layous K, Hult JR, Brousset A, Cotten P, Maurer S, Pietrucha ME, Acree M, Wrubel J, Johnson MO, Hecht F, Folkman S (2014). Randomized controlled trial of a positive affect intervention to reduce stress in people newly diagnosed with HIV; protocol and design for the IRISS study. OAJCT.

[24] Cohn MA, Pietrucha ME, Saslow LR, Hult JR, Moskowitz JT (2014). An online positive affect skills intervention reduces depression in adults with type 2 diabetes. J Posit Psychol.

[25] Addington EL, Cheung EO, Bassett SM, Kwok I, Schuette SA, Shiu E, Yang D, Cohn MA, Leykin Y, Saslow LR, Moskowitz JT (2019). The MARIGOLD study: feasibility and enhancement of an online intervention to improve emotion regulation in people with elevated depressive symptoms. J Affect Disord.

[26] Cheung EO, Addington EL, Bassett SM, Schuette SA, Shiu EW, Cohn MA, Leykin Y, Saslow LR, Moskowitz JT (2018). A self-paced, web-based, positive emotion skills intervention for reducing symptoms of depression: protocol for development and pilot testing of MARIGOLD. JMIR Res Protoc.

[27] Moskowitz JT, Addington EL, Shiu E, Bassett SM, Schuette S, Kwok I, Freedman ME, Leykin Y, Saslow LR, Cohn MA, Cheung EO (2021). Facilitator contact, discussion boards, and virtual badges as adherence enhancements to a web-based, self-guided, positive psychological intervention for depression: randomized controlled trial. J Med Internet Res.

[28] Bassett SM, Cohn M, Cotten P, Kwok I, Moskowitz JT (2019). Feasibility and acceptability of an online positive affect intervention for those living with comorbid HIV depression. AIDS Behav.

[29] Salsman JM, McLouth LE, Cohn M, Tooze JA, Sorkin M, Moskowitz JT (2020). A web-based, positive emotion skills intervention for enhancing posttreatment psychological well-being in young adult cancer survivors (EMPOWER): protocol for a single-arm feasibility Trial. JMIR Res Protoc.

[30] Addington EL, Cummings P, Jackson K, Yang D, Moskowitz JT (2023). Exploring retention, usage, and efficacy of web-based delivery of positive emotion regulation skills during the COVID-19 pandemic. Affect Sci.

[31] Gandy M, Pang STY, Scott AJ, Heriseanu AI, Bisby MA, Dudeney J, Karin E, Titov N, Dear BF (2022). Internet-delivered cognitive and behavioural based interventions for adults with chronic pain: a systematic review and meta-analysis of randomized controlled trials. Pain.

[32] Fredrickson BL (2001). The role of positive emotions in positive psychology. The broaden-and-build theory of positive emotions. Am Psychol.

[33] Fredrickson BL (2013). Positive emotions broaden and build. Adv Exp Soc Psychol.

[34] Moskowitz JT, Addington EL, Cheung EO (2019). Positive psychology and health: well-being interventions in the context of illness. Gen Hosp Psychiatry.

[35] Folkman S (1997). Positive psychological states and coping with severe stress. Soc Sci Med.

[36] Pressman SD, Cohen S (2005). Does positive affect influence health?. Psychol Bull.

[37] Layous K, Chancellor J, Lyubomirsky S (2014). Positive activities as protective factors against mental health conditions. J Abnorm Psychol.

[38] Lyubomirsky S, Layous K (2013). How do simple positive activities increase well-being?. Curr Dir Psychol Sci.

[39] Ong AD, Wilcox KT, Moskowitz JT, Wethington E, Addington EL, Sanni MO, Kim P, Reid MC (2023). Feasibility, acceptability, and preliminary efficacy of a positive affect skills intervention for adults with fibromyalgia. Innovation Aging.

[40] Bernardy K, Klose P, Welsch P, Häuser W (2019). Efficacy, acceptability and safety of internet-delivered psychological therapies for fibromyalgia syndrome: a systematic review and meta-analysis of randomized controlled trials. Eur J Pain.

[41] Donisi V, de Lucia A, Pasini I, Gandolfi M, Schweiger V, del Piccolo Lidia, Perlini Cinzia (2023). e-Health interventions targeting pain-related psychological variables in fibromyalgia: a systematic review. Healthcare (Basel).

[42] Ong AD, Moskowitz JT, Wethington E, Addington EL, Sanni M, Goktas S, Sluys E, Swong S, Kim P, Reid MC (2022). Lessons in Affect Regulation to Keep Stress and Pain UndeR control (LARKSPUR): design of a randomized controlled trial to increase positive affect in middle-aged and older adults with fibromyalgia. Contemp Clin Trials.

[43] Wolfe F, Clauw DJ, Fitzcharles MA, Goldenberg DL, Häuser W, Katz RS, Mease P, Russell AS, Russell IJ, Winfield JB (2011). Fibromyalgia criteria and severity scales for clinical and epidemiological studies: a modification of the ACR preliminary diagnostic criteria for fibromyalgia. J Rheumatol.

[44] Callahan CM, Unverzagt FW, Hui SL, Perkins AJ, Hendrie HC (2002). Six-item screener to identify cognitive impairment among potential subjects for clinical research. Med Care.

[45] Krause N (1988). Positive life events and depressive symptoms in older adults. Behav Med.

[46] Lewinsohn PM, Amenson CS (1978). Some relations between pleasant and unpleasant mood-related events and depression. J Abnorm Psychol.

[47] Bryant FB (2006). A four‐factor model of perceived control: avoiding, coping, obtaining, and savoring. J Pers.

[48] Langston CA (1994). Capitalizing on and coping with daily-life events: expressive responses to positive events. J Pers Soc Psychol.

[49] Taylor SE, Kemeny ME, Aspinwall LG, Schneider SG, Rodriguez R, Herbert M (1992). Optimism, coping, psychological distress, and high-risk sexual behavior among men at risk for acquired immunodeficiency syndrome (AIDS). J Pers Soc Psychol.

[50] Taylor SE, Lobel M (1989). Social comparison activity under threat: downward evaluation and upward contacts. Psychol Rev.

[51] Manos RC, Kanter JW, Busch AM (2010). A critical review of assessment strategies to measure the behavioral activation model of depression. Clin Psychol Rev.

[52] Moskowitz JT, Hult JR, Bussolari C, Acree M (2009). What works in coping with HIV? A meta-analysis with implications for coping with serious illness. Psychol Bull.

[53] Grossman P, Tiefenthaler-Gilmer U, Raysz A, Kesper U (2007). Mindfulness training as an intervention for fibromyalgia: evidence of postintervention and 3-year follow-up benefits in well-being. Psychother Psychosom.

[54] Kabat-Zinn J (1982). An outpatient program in behavioral medicine for chronic pain patients based on the practice of mindfulness meditation: theoretical considerations and preliminary results. Gen Hosp Psychiatry.

[55] Moskowitz JT, Folkman S, Collette L, Vittinghoff E (1996). Coping and mood during aids-related caregiving and bereavement. Ann Behav Med.

[56] Emmons RA, Crumpler CA (2000). Gratitude as a human strength: appraising the evidence. J Soc Clin Psychol.

[57] Fredrickson BL, Emmons RA, McCullough ME (2004). Gratitude, like other positive emotions, broadens and builds. The Psychology of Gratitude.

[58] Brown SL, Nesse RM, Vinokur AD, Smith DM (2003). Providing social support may be more beneficial than receiving it: results from a prospective study of mortality. Psychol Sci.

[59] Penner LA, Dovidio JF, Piliavin JA, Schroeder DA (2005). Prosocial behavior: multilevel perspectives. Annu Rev Psychol.

[60] Fredrickson BL, Tugade MM, Waugh CE, Larkin GR (2003). What good are positive emotions in crises? A prospective study of resilience and emotions following the terrorist attacks on the United States on September 11th, 2001. J Pers Soc Psychol.

[61] Ong AD, Zautra AJ, Reid MC (2010). Psychological resilience predicts decreases in pain catastrophizing through positive emotions. Psychol Aging.

[62] Depaoli S, van de Schoot R (2017). Improving transparency and replication in Bayesian statistics: the WAMBS-checklist. Psychol Methods.

[63] Kruschke JK (2015). Doing Bayesian Data Analysis: A Tutorial with R, JAGS, and Stan.

[64] Kruschke JK (2018). Rejecting or accepting parameter values in Bayesian estimation. Adv Methods Pract Psychol Sci.

[65] Makowski DS, Ben-Shachar M, Lüdecke D (2019). bayestestR: describing effects and their uncertainty, existence and significance within the bayesian Framework. JOSS.

[66] Makowski D, Ben-Shachar MS, Chen SHA, Lüdecke D (2019). Indices of effect existence and significance in the Bayesian framework. Front Psychol.

[67] Goldstein H, Kounali D, Robinson A (2008). Modelling measurement errors and category misclassifications in multilevel models. Stat Modell.

[68] Bollen KA (1989). Structural Equations with Latent Variables.

[69] Loken E, Gelman A (2017). Measurement error and the replication crisis. Science.

[70] Williams LJ, Hazer JT (1986). Antecedents and consequences of satisfaction and commitment in turnover models: a reanalysis using latent variable structural equation methods. J Appl Psychol.

[71] Cronbach LJ (1951). Coefficient alpha and the internal structure of tests. Psychometrika.

[72] Singer JD, Willett JB (2003). Applied Longitudinal Data Analysis: Modeling Change and Event Occurrence.

[73] Gelman A, Carlin JB, Stern HS, Dunson DB, Vehtari A, Rubin DB (2013). Bayesian Data Analysis.

[74] Bürkner PC (2017). brms: an R package for Bayesian multilevel models using Stan. J Stat Software.

[75] Mader N, Arslan RC, Schmukle SC, Rohrer JM (2023). Emotional (in)stability: neuroticism is associated with increased variability in negative emotion after all. Proc Natl Acad Sci U S A.

[76] Curran PJ, Bauer DJ (2011). The disaggregation of within-person and between-person effects in longitudinal models of change. Annu Rev Psychol.

[77] Moher D, Hopewell S, Schulz KF, Montori V, Gøtzsche PC, Devereaux PJ, Elbourne D, Egger M, Altman DG (2010). CONSORT 2010 explanation and elaboration: updated guidelines for reporting parallel group randomised trials. BMJ.

[78] Ong AD (2010). Pathways linking positive emotion and health in later life. Curr Dir Psychol Sci.

[79] Andersson G, Cuijpers P (2009). Internet-based and other computerized psychological treatments for adult depression: a meta-analysis. Cogn Behav Ther.

[80] Bender JL, Radhakrishnan A, Diorio C, Englesakis M, Jadad AR (2011). Can pain be managed through the internet? A systematic review of randomized controlled trials. Pain.

[81] Eccleston C, Blyth FM, Dear BF, Fisher EA, Keefe FJ, Lynch ME, Palermo TM, Reid MC, Williams ACDC (2020). Managing patients with chronic pain during the COVID-19 outbreak: considerations for the rapid introduction of remotely supported (eHealth) pain management services. Pain.

[82] Tauben DJ, Langford DJ, Sturgeon JA, Rundell SD, Towle C, Bockman C, Nicholas M (2020). Optimizing telehealth pain care after COVID-19. Pain.

[83] Kashdan TB, Rottenberg J (2010). Psychological flexibility as a fundamental aspect of health. Clin Psychol Rev.

[84] Lee S, Koffer RE, Sprague BN, Charles ST, Ram N, Almeida DM (2018). Activity diversity and its associations with psychological well-being across adulthood. J Gerontol B Psychol Sci Soc Sci.

[85] Ong AD, Lee S (2024). Variety in pleasant activities is associated with improved mental health: evidence from two national samples of U.S. adults. Affect Sci.

