# Supplementary material for: Targeting Daily Positive Events to Improve Emotional and Functional Well-Being in Adults With Fibromyalgia: Insights From the LARKSPUR Randomized Controlled Trial
Source: J Med Internet Res. 2024 Dec 10;26:e54678. doi: 10.2196/54678 (PMC11668987; doi:10.2196/54678)
Supplement: Multimedia Appendix 1 [file jmir_v26i1e54678_app1.pdf]

# CONSORT-EHEALTH (V 1.6.1) - Submission/Publication Form

The CONSORT-EHEALTH checklist is intended for authors of randomized trials evaluating web-based and Internet-based applications/interventions, including mobile interventions, electronic games (incl multiplayer games), social media, certain telehealth applications, and other interactive and/or networked electronic applications. Some of the items (e.g. all subitems under item 5 - description of the intervention) may also be applicable for other study designs.

The goal of the CONSORT EHEALTH checklist and guideline is to be
a) a guide for reporting for authors of RCTs,
b) to form a basis for appraisal of an ehealth trial (in terms of validity)

CONSORT-EHEALTH items/subitems are MANDATORY reporting items for studies published in the Journal of Medical Internet Research and other journals / scientific societies endorsing the checklist.

Items numbered 1., 2., 3., 4a., 4b etc are original CONSORT or CONSORT-NPT (non-pharmacologic treatment) items.
Items with Roman numerals (i., ii, iii, iv etc.) are CONSORT-EHEALTH extensions/clarifications.

As the CONSORT-EHEALTH checklist is still considered in a formative stage, we would ask that you also RATE ON A SCALE OF 1-5 how important/useful you feel each item is FOR THE PURPOSE OF THE CHECKLIST and reporting guideline (optional).

Mandatory reporting items are marked with a red *.
In the textboxes, either copy & paste the relevant sections from your manuscript into this form - please include any quotes from your manuscript in QUOTATION MARKS,
or answer directly by providing additional information not in the manuscript, or elaborating on why the item was not relevant for this study.

YOUR ANSWERS WILL BE PUBLISHED AS  A SUPPLEMENTARY FILE TO YOUR PUBLICATION IN JMIR AND ARE CONSIDERED PART OF YOUR PUBLICATION (IF ACCEPTED).
Please fill in these questions diligently. Information will not be copyedited, so please use proper spelling and grammar, use correct capitalization, and avoid abbreviations.

DO NOT FORGET TO SAVE AS PDF _AND_ CLICK THE SUBMIT BUTTON SO YOUR ANSWERS ARE IN OUR DATABASE !!!

Citation Suggestion (if you append the pdf as Appendix we suggest to cite this paper in the caption):
Eysenbach G, CONSORT-EHEALTH Group

CONSORT-EHEALTH: Improving and Standardizing Evaluation Reports of Web-based and Mobile Health Interventions
J Med Internet Res 2011;13(4):e126
URL: http://www.jmir.org/2011/4/e126/
doi: 10.2196/jmir.1923
PMID: 22209829

ado4happy@gmail.com Switch account

Not shared

Draft saved

* Indicates required question

Your name *

First Last

Anthony Ong

Primary Affiliation (short), City, Country *

University of Toronto, Toronto, Canada

Cornell University, Ithaca, USA

Your e-mail address *

abc@gmail.com

anthony.ong@cornell.edu

Title of your manuscript *

Provide the (draft) title of your manuscript.

Targeting Daily Positive Experiences to Improve Emotional and Functional Well-being in Adults with Fibromyalgia: Insights from the LARKSPUR Randomized Controlled Trial

Name of your App/Software/Intervention *

If there is a short and a long/alternate name, write the short name first and add the long name in brackets.

Lessons in Affect Regulation to Keep Stress ar

Evaluated Version (if any)

e.g. "V1", "Release 2017-03-01", "Version 2.0.27913"

Your answer

Language(s) *

What language is the intervention/app in? If multiple languages are available, separate by comma (e.g. "English, French")

English

URL of your Intervention Website or App

e.g. a direct link to the mobile app on app in appstore (itunes, Google Play), or URL of the website. If the intervention is a DVD or hardware, you can also link to an Amazon page.

Your answer

URL of an image/screenshot (optional)

Your answer

Accessibility *

Can an enduser access the intervention presently?

- ( ) access is free and open
- (x) access only for special usergroups, not open
- ( ) access is open to everyone, but requires payment/subscription/in-app purchases
- ( ) app/intervention no longer accessible
- ( ) Other:

Primary Medical Indication/Disease/Condition *

e.g. "Stress", "Diabetes", or define the target group in brackets after the condition, e.g. "Autism (Parents of children with)", "Alzheimers (Informal Caregivers of)"

Fibromyalgia Syndrome (FMS)

Primary Outcomes measured in trial *

comma-separated list of primary outcomes reported in the trial

positive affect, negative affect, pain, fatigue

Secondary/other outcomes

Are there any other outcomes the intervention is expected to affect?

Your answer

Recommended "Dose" *

What do the instructions for users say on how often the app should be used?

- (x) Approximately Daily
- ( ) Approximately Weekly
- ( ) Approximately Monthly
- ( ) Approximately Yearly
- ( ) "as needed"
- ( ) Other:

Approx. Percentage of Users (starters) still using the app as recommended after 3 months *

- ( ) unknown / not evaluated
- ( ) 0-10%
- ( ) 11-20%
- ( ) 21-30%
- ( ) 31-40%
- ( ) 41-50%
- ( ) 51-60%
- (x) 61-70%
- ( ) 71%-80%
- ( ) 81-90%
- ( ) 91-100%
- ( ) Other:

Overall, was the app/intervention effective? *

- (x) yes: all primary outcomes were significantly better in intervention group vs control
- ( ) partly: SOME primary outcomes were significantly better in intervention group vs control
- ( ) no statistically significant difference between control and intervention
- ( ) potentially harmful: control was significantly better than intervention in one or more outcomes
- ( ) inconclusive: more research is needed
- ( ) Other:

Article Preparation Status/Stage *

At which stage in your article preparation are you currently (at the time you fill in this form)

- ( ) not submitted yet - in early draft status
- ( ) not submitted yet - in late draft status, just before submission
- ( ) submitted to a journal but not reviewed yet
- (x) submitted to a journal and after receiving initial reviewer comments
- ( ) submitted to a journal and accepted, but not published yet
- ( ) published
- ( ) Other:

Journal *

If you already know where you will submit this paper (or if it is already submitted), please provide the journal name (if it is not JMIR, provide the journal name under "other")

- ( ) not submitted yet / unclear where I will submit this
- (•) Journal of Medical Internet Research (JMIR)
- ( ) JMIR mHealth and UHealth
- ( ) JMIR Serious Games
- ( ) JMIR Mental Health
- ( ) JMIR Public Health
- ( ) JMIR Formative Research
- ( ) Other JMIR sister journal
- ( ) Other:

Is this a full powered effectiveness trial or a pilot/feasibility trial? *

- (•) Pilot/feasibility
- ( ) Fully powered

Manuscript tracking number *

If this is a JMIR submission, please provide the manuscript tracking number under "other" (The ms tracking number can be found in the submission acknowledgement email, or when you login as author in JMIR. If the paper is already published in JMIR, then the ms tracking number is the four-digit number at the end of the DOI, to be found at the bottom of each published article in JMIR)

- ○ no ms number (yet) / not (yet) submitted to / published in JMIR
- ◉ Other: JMIR ms#54678

TITLE AND ABSTRACT

1a) TITLE: Identification as a randomized trial in the title

1a) Does your paper address CONSORT item 1a? *

I.e does the title contain the phrase "Randomized Controlled Trial"? (if not, explain the reason under "other")

- ◉ yes
- ○ Other:

### 1a-i) Identify the mode of delivery in the title

Identify the mode of delivery. Preferably use “web-based” and/or “mobile” and/or “electronic game” in the title. Avoid ambiguous terms like “online”, “virtual”, “interactive”. Use “Internet-based” only if Intervention includes non-web-based Internet components (e.g. email), use “computer-based” or “electronic” only if offline products are used. Use “virtual” only in the context of “virtual reality” (3-D worlds). Use “online” only in the context of “online support groups”. Complement or substitute product names with broader terms for the class of products (such as “mobile” or “smart phone” instead of “iphone”), especially if the application runs on different platforms.

| | 1 | 2 | 3 | 4 | 5 | |
|---|---|---|---|---|---|---|
| subitem not at all important | ○ | ○ | ◉ | ○ | ○ | essential |

Clear selection

### Does your paper address subitem 1a-i? *

Copy and paste relevant sections from manuscript title (include quotes in quotation marks "like this" to indicate direct quotes from your manuscript), or elaborate on this item by providing additional information not in the ms, or briefly explain why the item is not applicable/relevant for your study

This randomized controlled trial investigated the efficacy of a web-based positive affect regulation intervention, Lessons in Affect Regulation to Keep Stress and Pain UndeR control (LARKSPUR), in enhancing emotional and functional well-being among adults with fibromyalgia syndrome (FMS).

Web-based programs can overcome barriers to in-person treatment while providing effective tools to boost positive experiences.

To address this gap, the current study tested a web-based program that integrates prior theoretical work on positive emotions [33–35], stress and coping [36,37], and PE [38,39].

1a-ii) Non-web-based components or important co-interventions in title

Mention non-web-based components or important co-interventions in title, if any (e.g., “with telephone support”).

| | 1 | 2 | 3 | 4 | 5 | |
|---|---|---|---|---|---|---|
| subitem not at all important | (●) | ( ) | ( ) | ( ) | ( ) | essential |

Clear selection

Does your paper address subitem 1a-ii?

Copy and paste relevant sections from manuscript title (include quotes in quotation marks "like this" to indicate direct quotes from your manuscript), or elaborate on this item by providing additional information not in the ms, or briefly explain why the item is not applicable/relevant for your study

Non-web based components are not important in this study

1a-iii) Primary condition or target group in the title

Mention primary condition or target group in the title, if any (e.g., “for children with Type I Diabetes”) Example: A Web-based and Mobile Intervention with Telephone Support for Children with Type I Diabetes: Randomized Controlled Trial

| | 1 | 2 | 3 | 4 | 5 | |
|---|---|---|---|---|---|---|
| subitem not at all important | ( ) | ( ) | ( ) | ( ) | (●) | essential |

Clear selection

Does your paper address subitem 1a-iii? *

Copy and paste relevant sections from manuscript title (include quotes in quotation marks "like this" to indicate direct quotes from your manuscript), or elaborate on this item by providing additional information not in the ms, or briefly explain why the item is not applicable/relevant for your study

Targeting Daily Positive Experiences to Improve Emotional and Functional Well-being in Adults with Fibromyalgia: Insights from the LARKSPUR Intervention

1b) ABSTRACT: Structured summary of trial design, methods, results, and conclusions

NPT extension: Description of experimental treatment, comparator, care providers, centers, and blinding status.

1b-i) Key features/functionalities/components of the intervention and comparator in the METHODS section of the ABSTRACT

Mention key features/functionalities/components of the intervention and comparator in the abstract. If possible, also mention theories and principles used for designing the site. Keep in mind the needs of systematic reviewers and indexers by including important synonyms. (Note: Only report in the abstract what the main paper is reporting. If this information is missing from the main body of text, consider adding it)

| | 1 | 2 | 3 | 4 | 5 | |
|---|---|---|---|---|---|---|
| subitem not at all important | ○ | ○ | ○ | ◉ | ○ | essential |

Clear selection

Does your paper address subitem 1b-i? *

Copy and paste relevant sections from the manuscript abstract (include quotes in quotation marks "like this" to indicate direct quotes from your manuscript), or elaborate on this item by providing additional information not in the ms, or briefly explain why the item is not applicable/relevant for your study

Ninety-five FMS participants aged 50 and older (94% female) were randomized to one of two fully-automated conditions: (1) LARKSPUR (n = 49) or (2) emotion reporting/attention control (n = 46).

1b-ii) Level of human involvement in the METHODS section of the ABSTRACT

Clarify the level of human involvement in the abstract, e.g., use phrases like “fully automated” vs. “therapist/nurse/care provider/physician-assisted” (mention number and expertise of providers involved, if any). (Note: Only report in the abstract what the main paper is reporting. If this information is missing from the main body of text, consider adding it)

| | 1 | 2 | 3 | 4 | 5 | |
|---|---|---|---|---|---|---|
| subitem not at all important | ○ | ○ | ○ | ◉ | ○ | essential |

Clear selection

Does your paper address subitem 1b-ii?

Copy and paste relevant sections from the manuscript abstract (include quotes in quotation marks "like this" to indicate direct quotes from your manuscript), or elaborate on this item by providing additional information not in the ms, or briefly explain why the item is not applicable/relevant for your study

Ninety-five FMS participants aged 50 and older (94% female) were randomized to one of two fully-automated conditions: (1) LARKSPUR (n = 49) or (2) emotion reporting/attention control (n = 46).

### 1b-iii) Open vs. closed, web-based (self-assessment) vs. face-to-face assessments in the METHODS section of the ABSTRACT

Mention how participants were recruited (online vs. offline), e.g., from an open access website or from a clinic or a closed online user group (closed usergroup trial), and clarify if this was a purely web-based trial, or there were face-to-face components (as part of the intervention or for assessment). Clearly say if outcomes were self-assessed through questionnaires (as common in web-based trials). Note: In traditional offline trials, an open trial (open-label trial) is a type of clinical trial in which both the researchers and participants know which treatment is being administered. To avoid confusion, use “blinded” or “unblinded” to indicated the level of blinding instead of “open”, as “open” in web-based trials usually refers to “open access” (i.e. participants can self-enrol). (Note: Only report in the abstract what the main paper is reporting. If this information is missing from the main body of text, consider adding it)

| | 1 | 2 | 3 | 4 | 5 | |
|---|---|---|---|---|---|---|
| subitem not at all important | ○ | ○ | ◉ | ○ | ○ | essential |

Clear selection

### Does your paper address subitem 1b-iii?

Copy and paste relevant sections from the manuscript abstract (include quotes in quotation marks "like this" to indicate direct quotes from your manuscript), or elaborate on this item by providing additional information not in the ms, or briefly explain why the item is not applicable/relevant for your study

At posttreatment and 1-month follow-up, participants completed 7 consecutive end-of-day online reports capturing positive events, pain, fatigue, positive affect, and negative affect.

1b-iv) RESULTS section in abstract must contain use data

Report number of participants enrolled/assessed in each group, the use/uptake of the intervention (e.g., attrition/adherence metrics, use over time, number of logins etc.), in addition to primary/secondary outcomes. (Note: Only report in the abstract what the main paper is reporting. If this information is missing from the main body of text, consider adding it)

| | 1 | 2 | 3 | 4 | 5 | |
|---|---|---|---|---|---|---|
| subitem not at all important | ○ | ○ | ○ | ◉ | ○ | essential |

Clear selection

Does your paper address subitem 1b-iv?

Copy and paste relevant sections from the manuscript abstract (include quotes in quotation marks "like this" to indicate direct quotes from your manuscript), or elaborate on this item by providing additional information not in the ms, or briefly explain why the item is not applicable/relevant for your study

Ninety-five FMS participants aged 50 and older (94% female) were randomized to one of two fully-automated conditions: (1) LARKSPUR (n = 49) or (2) emotion reporting/attention control (n = 46). At posttreatment and 1-month follow-up, participants completed 7 consecutive end-of-day online reports capturing positive events, pain, fatigue, positive affect, and negative affect.

### 1b-v) CONCLUSIONS/DISCUSSION in abstract for negative trials

Conclusions/Discussions in abstract for negative trials: Discuss the primary outcome - if the trial is negative (primary outcome not changed), and the intervention was not used, discuss whether negative results are attributable to lack of uptake and discuss reasons. (Note: Only report in the abstract what the main paper is reporting. If this information is missing from the main body of text, consider adding it)

| | 1 | 2 | 3 | 4 | 5 | |
|---|---|---|---|---|---|---|
| subitem not at all important | (●) | ○ | ○ | ○ | ○ | essential |

Clear selection

### Does your paper address subitem 1b-v?

Copy and paste relevant sections from the manuscript abstract (include quotes in quotation marks "like this" to indicate direct quotes from your manuscript), or elaborate on this item by providing additional information not in the ms, or briefly explain why the item is not applicable/relevant for your study

Study results did not find evidence of negative trials

## INTRODUCTION

### 2a) In INTRODUCTION: Scientific background and explanation of rationale

2a-i) Problem and the type of system/solution

Describe the problem and the type of system/solution that is object of the study: intended as stand-alone intervention vs. incorporated in broader health care program? Intended for a particular patient population? Goals of the intervention, e.g., being more cost-effective to other interventions, replace or complement other solutions? (Note: Details about the intervention are provided in “Methods” under 5)

| | 1 | 2 | 3 | 4 | 5 | |
|---|---|---|---|---|---|---|
| subitem not at all important | ○ | ○ | ○ | ◉ | ○ | essential |

Clear selection

Does your paper address subitem 2a-i? *

Copy and paste relevant sections from the manuscript (include quotes in quotation marks "like this" to indicate direct quotes from your manuscript), or elaborate on this item by providing additional information not in the ms, or briefly explain why the item is not applicable/relevant for your study

Chronic pain is a major public health problem affecting millions of people worldwide and imposing significant burdens on individuals' well-being and quality of life. Among chronic pain conditions, fibromyalgia syndrome (FMS) is one of the most prevalent and challenging to treat. FMS involves widespread musculoskeletal pain, fatigue, and debilitating symptoms that impair physical and psychological functioning [1–3]. While pharmacological treatments provide partial relief for some individuals, non-pharmacologic approaches are often needed to improve well-being [4]. Consequently, innovative interventions are required that address the multifaceted impacts of FMS on daily life.

2a-ii) Scientific background, rationale: What is known about the (type of) system

Scientific background, rationale: What is known about the (type of) system that is the object of the study (be sure to discuss the use of similar systems for other conditions/diagnoses, if appropiate), motivation for the study, i.e. what are the reasons for and what is the context for this specific study, from which stakeholder viewpoint is the study performed, potential impact of findings [2]. Briefly justify the choice of the comparator.

| | 1 | 2 | 3 | 4 | 5 | |
|---|---|---|---|---|---|---|
| subitem not at all important | ○ | ○ | ○ | ◉ | ○ | essential |

Clear selection

Does your paper address subitem 2a-ii? *

Copy and paste relevant sections from the manuscript (include quotes in quotation marks "like this" to indicate direct quotes from your manuscript), or elaborate on this item by providing additional information not in the ms, or briefly explain why the item is not applicable/relevant for your study

Chronic pain is a major public health problem affecting millions of people worldwide and imposing significant burdens on individuals' well-being and quality of life. Among chronic pain conditions, fibromyalgia syndrome (FMS) is one of the most prevalent and challenging to treat. FMS involves widespread musculoskeletal pain, fatigue, and debilitating symptoms that impair physical and psychological functioning [1–3]. While pharmacological treatments provide partial relief for some individuals, non-pharmacologic approaches are often needed to improve well-being [4]. Consequently, innovative interventions are required that address the multifaceted impacts of FMS on daily life.

In recent years, positive psychological interventions have emerged as a promising approach to promote well-being and enhance mental health outcomes [5–7]. Integrating daily positive activities into chronic pain treatment has shown potential in fostering resilience and improving overall well-being [8,9]. Daily positive events (PE)—small, meaningful experiences that increase positive emotions [10,11]—may be especially beneficial for individuals with FMS. First, cultivating positive emotions like joy, gratitude, and contentment through daily positive events could improve emotional well-being, as these tend to be deficient in this population [12,13]. Second, actively seeking and savoring positive experiences may instill a greater sense of control and agency over one's life, counterbalancing feelings of helplessness and hopelessness common in chronic pain [14,15]. Third, pleasant daily activities could facilitate social connection and support, which are essential given individuals with FMS often experience isolation due to their condition [16,17]. Finally, engagement in pleasant activities and PE may facilitate healthy habits and routines, promoting sustained well-being and improvements in daily functioning [18,19].
Internet-delivered positive psychological interventions represent a promising approach to enhance well-being among those affecting by chonric pain, including those with FMS. Web-based programs can overcome barriers to in-person treatment while providing effective tools to boost positive experiences. One such intervention has been widely tested in multiple studies with more than 1,000 participants (ages 16 to 78) coping with varied life stressors, from diagnosis with a serious illness to daily stress [20–23]. The program has been implemented in person (individually and in groups), and most recently, has been delivered online as a self-guided program for individuals with diabetes [24], depression [25–27], HIV [28], cancer [29,30], and for the general public during the COVID-19 pandemic [31]. However, research is lacking on how to optimally design such interventions specifically for aging adults with FMS [32].

2b) In INTRODUCTION: Specific objectives or hypotheses

Does your paper address CONSORT subitem 2b? *

Copy and paste relevant sections from the manuscript (include quotes in quotation marks "like this" to indicate direct quotes from your manuscript), or elaborate on this item by providing additional information not in the ms, or briefly explain why the item is not applicable/relevant for your study

Consistent with the Positive Pathways to Health model [35], we hypothesized that participants randomized to LARKSPUR would show greater enhancements in daily positive event-related affective well-being, pain, and fatigue compared to participants in the emotion reporting/attention control condition.

METHODS

3a) Description of trial design (such as parallel, factorial) including allocation ratio

Does your paper address CONSORT subitem 3a? *

Copy and paste relevant sections from the manuscript (include quotes in quotation marks "like this" to indicate direct quotes from your manuscript), or elaborate on this item by providing additional information not in the ms, or briefly explain why the item is not applicable/relevant for your study

LARKSPUR Intervention. Individuals randomized to LARKSPUR received skills training to increase PA. The web-based intervention was self-guided and targeted eight PA skills over five weekly learning modules. The eight skills included, (1) noticing positive events [46,47]; (2) savoring positive events [48,49]; (3) identifying personal strengths [50,51]; (4) behavioral activation to set and work toward attainable goals [52,53]; (5) mindfulness [54,55]; (6) positive reappraisal [36,56]; (7) gratitude [57,58]; and (8) acts of kindness [59,60]. Each module consisted of a video introduction, interactive exercises, examples, and homework assignments. Participants practiced skills daily and reported experiences on the online platform.

Control Program. Control participants completed daily online emotion reports, rating positive and negative emotions over the past 24 hours on a 5-point Likert scale. This control condition was designed to match the LARKSPUR group in terms of online contact, attention to emotional states, and study duration, without providing any specific skills or strategies to enhance positive affect or cope with pain. Previous studies have used similar emotion reporting as a control condition for positive psychology interventions [35].
Participants in both arms were assessed at baseline, at 8 weeks (post-intervention), and 1-month follow-up. In addition, before (baseline) and after the intervention (post) and at 1-month follow-up, participants completed a 7-day burst of online daily assessments of positive events, positive affect (PA) and negative affect (NA), pain intensity, and fatigue.

3b) Important changes to methods after trial commencement (such as eligibility criteria), with reasons

Does your paper address CONSORT subitem 3b? *

Copy and paste relevant sections from the manuscript (include quotes in quotation marks "like this" to indicate direct quotes from your manuscript), or elaborate on this item by providing additional information not in the ms, or briefly explain why the item is not applicable/relevant for your study

The LARKSPUR pilot (Trial Registration: NCT04869345) is described elsewhere [43]. Briefly, we recruited participants who met the following criteria: (1) age ≥ 50 years, (2) access to Wi-Fi Internet connection, (3) English literacy via self-reports of fluency and reading and writing comprehension,, and (4) diagnosis of FMS based on the American College of Rheumatology (ACR) Fibromyalgia Symptom Severity Scale [44] and/or physician confirmation of FMS. Exclusions were (1) moderate or severe cognitive impairment (two or more errors on 6-item Mini-mental state examination [MMSE]) [45], (2) current behavioral treatment for chronic pain, or (3) enrollment in another chronic pain trial. Eligible and consenting participants were randomized to LARKSPUR or control.

3b-i) Bug fixes, Downtimes, Content Changes

Bug fixes, Downtimes, Content Changes: ehealth systems are often dynamic systems. A description of changes to methods therefore also includes important changes made on the intervention or comparator during the trial (e.g., major bug fixes or changes in the functionality or content) (5-iii) and other “unexpected events” that may have influenced study design such as staff changes, system failures/downtimes, etc. [2].

| | 1 | 2 | 3 | 4 | 5 | |
|---|---|---|---|---|---|---|
| subitem not at all important | (•) | ○ | ○ | ○ | ○ | essential |

Clear selection

Does your paper address subitem 3b-i?

Copy and paste relevant sections from the manuscript (include quotes in quotation marks "like this" to indicate direct quotes from your manuscript), or elaborate on this item by providing additional information not in the ms, or briefly explain why the item is not applicable/relevant for your study

There were not bug fixes, downtimes, or content changes to this study

4a) Eligibility criteria for participants

Does your paper address CONSORT subitem 4a? *

Copy and paste relevant sections from the manuscript (include quotes in quotation marks "like this" to indicate direct quotes from your manuscript), or elaborate on this item by providing additional information not in the ms, or briefly explain why the item is not applicable/relevant for your study

The LARKSPUR pilot (Trial Registration: NCT04869345) is described elsewhere [43]. Briefly, we recruited participants who met the following criteria: (1) age ≥ 50 years, (2) access to Wi-Fi Internet connection, (3) English literacy via self-reports of fluency and reading and writing comprehension,, and (4) diagnosis of FMS based on the American College of Rheumatology (ACR) Fibromyalgia Symptom Severity Scale [44] and/or physician confirmation of FMS. Exclusions were (1) moderate or severe cognitive impairment (two or more errors on 6-item Mini-mental state examination [MMSE]) [45], (2) current behavioral treatment for chronic pain, or (3) enrollment in another chronic pain trial. Eligible and consenting participants were randomized to LARKSPUR or control.

4a-i) Computer / Internet literacy

Computer / Internet literacy is often an implicit “de facto” eligibility criterion - this should be explicitly clarified.

| | 1 | 2 | 3 | 4 | 5 | |
|---|---|---|---|---|---|---|
| subitem not at all important | (•) | ( ) | ( ) | ( ) | ( ) | essential |

Clear selection

Does your paper address subitem 4a-i?

Copy and paste relevant sections from the manuscript (include quotes in quotation marks "like this" to indicate direct quotes from your manuscript), or elaborate on this item by providing additional information not in the ms, or briefly explain why the item is not applicable/relevant for your study

This is assumed "de facto" in the eligibility criteria

4a-ii) Open vs. closed, web-based vs. face-to-face assessments:

Open vs. closed, web-based vs. face-to-face assessments: Mention how participants were recruited (online vs. offline), e.g., from an open access website or from a clinic, and clarify if this was a purely web-based trial, or there were face-to-face components (as part of the intervention or for assessment), i.e., to what degree got the study team to know the participant. In online-only trials, clarify if participants were quasi-anonymous and whether having multiple identities was possible or whether technical or logistical measures (e.g., cookies, email confirmation, phone calls) were used to detect/prevent these.

| | 1 | 2 | 3 | 4 | 5 | |
|---|---|---|---|---|---|---|
| subitem not at all important | ( ) | ( ) | ( ) | (•) | ( ) | essential |

Clear selection

Does your paper address subitem 4a-ii? *

Copy and paste relevant sections from the manuscript (include quotes in quotation marks "like this" to indicate direct quotes from your manuscript), or elaborate on this item by providing additional information not in the ms, or briefly explain why the item is not applicable/relevant for your study

Participants were recruited from July 2021 to June 2022 through referrals from New York State practicing physicians, posted flyers throughout the New York Presbyterian Healthcare System, New York City-based senior centers, community centers, and online platforms (e.g., Facebook groups). Recruitment links were also posted on clinicaltrials.gov and emailed to potential participants via ResearchMatch, a national health volunteer research registry created by several U.S. based academic institutions and supported by the U.S. National Institutes of Health.

4a-iii) Information giving during recruitment

Information given during recruitment. Specify how participants were briefed for recruitment and in the informed consent procedures (e.g., publish the informed consent documentation as appendix, see also item X26), as this information may have an effect on user self-selection, user expectation and may also bias results.

| | 1 | 2 | 3 | 4 | 5 | |
|---|---|---|---|---|---|---|
| subitem not at all important | ○ | ○ | ○ | ● | ○ | essential |

Clear selection

Does your paper address subitem 4a-iii?

Copy and paste relevant sections from the manuscript (include quotes in quotation marks "like this" to indicate direct quotes from your manuscript), or elaborate on this item by providing additional information not in the ms, or briefly explain why the item is not applicable/relevant for your study

Eligible and consenting participants were randomized to LARKSPUR or control. The study was reviewed and approved by the Institutional Review Board (IRB) at Weill Cornell Medicine.

4b) Settings and locations where the data were collected

Does your paper address CONSORT subitem 4b? *

Copy and paste relevant sections from the manuscript (include quotes in quotation marks "like this" to indicate direct quotes from your manuscript), or elaborate on this item by providing additional information not in the ms, or briefly explain why the item is not applicable/relevant for your study

Participants were recruited from July 2021 to June 2022 through referrals from New York State practicing physicians, posted flyers throughout the New York Presbyterian Healthcare System, New York City-based senior centers, community centers, and online platforms (e.g., Facebook groups). Recruitment links were also posted on clinicaltrials.gov and emailed to potential participants via ResearchMatch, a national health volunteer research registry created by several U.S. based academic institutions and supported by the U.S. National Institutes of Health.

4b-i) Report if outcomes were (self-)assessed through online questionnaires

Clearly report if outcomes were (self-)assessed through online questionnaires (as common in web-based trials) or otherwise.

| | 1 | 2 | 3 | 4 | 5 | |
|---|---|---|---|---|---|---|
| subitem not at all important | ○ | ○ | ○ | ○ | ● | essential |

Clear selection

Does your paper address subitem 4b-i? *

Copy and paste relevant sections from the manuscript (include quotes in quotation marks "like this" to indicate direct quotes from your manuscript), or elaborate on this item by providing additional information not in the ms, or briefly explain why the item is not applicable/relevant for your study

Positive events were assessed with 5 items asking participants to report whether the following positive events had occurred in the past 24 hours: (a) positive interpersonal interaction, (b) positive experience at work, school, or at a volunteer position, (c) positive experience at home, (d) network positive event (i.e., positive event experienced by a close friend or relative), and (e) any other positive event [10].

Positive and negative affect were assessed using the 20-item modified Differential Emotions Scale (mDES), which asked respondents to rate how often they felt 10 positive (e.g., amusement, gratitude, joy) and 10 negative (e.g., anger, guilt, sadness) emotions during the past 24 hours [61]. Items were rated on a 5-point Likert-type scale ranging from 0 ("never") to 4 ("most of the time").

Pain intensity and fatigue were each measured using a single item with a rating scale from 0 (" no pain / fatigue") to 10 ("as much pain / fatigue as could be").

4b-ii) Report how institutional affiliations are displayed

Report how institutional affiliations are displayed to potential participants [on ehealth media], as affiliations with prestigious hospitals or universities may affect volunteer rates, use, and reactions with regards to an intervention.(Not a required item – describe only if this may bias results)

| | 1 | 2 | 3 | 4 | 5 | |
|---|---|---|---|---|---|---|
| subitem not at all important | (●) | ○ | ○ | ○ | ○ | essential |

Clear selection

Does your paper address subitem 4b-ii?

Copy and paste relevant sections from the manuscript (include quotes in quotation marks "like this" to indicate direct quotes from your manuscript), or elaborate on this item by providing additional information not in the ms, or briefly explain why the item is not applicable/relevant for your study

This is not relevant to this study

5) The interventions for each group with sufficient details to allow replication, including how and when they were actually administered

5-i) Mention names, credential, affiliations of the developers, sponsors, and owners

Mention names, credential, affiliations of the developers, sponsors, and owners [6] (if authors/evaluators are owners or developer of the software, this needs to be declared in a “Conflict of interest” section or mentioned elsewhere in the manuscript).

| | 1 | 2 | 3 | 4 | 5 | |
|---|---|---|---|---|---|---|
| subitem not at all important | (●) | ( ) | ( ) | ( ) | ( ) | essential |

Clear selection

Does your paper address subitem 5-i?

Copy and paste relevant sections from the manuscript (include quotes in quotation marks "like this" to indicate direct quotes from your manuscript), or elaborate on this item by providing additional information not in the ms, or briefly explain why the item is not applicable/relevant for your study

this information is not relevant for this study

### 5-ii) Describe the history/development process

Describe the history/development process of the application and previous formative evaluations (e.g., focus groups, usability testing), as these will have an impact on adoption/use rates and help with interpreting results.

| | 1 | 2 | 3 | 4 | 5 | |
|---|---|---|---|---|---|---|
| subitem not at all important | (•) | ○ | ○ | ○ | ○ | essential |

Clear selection

### Does your paper address subitem 5-ii?

Copy and paste relevant sections from the manuscript (include quotes in quotation marks "like this" to indicate direct quotes from your manuscript), or elaborate on this item by providing additional information not in the ms, or briefly explain why the item is not applicable/relevant for your study

this information is not relevant for this study

### 5-iii) Revisions and updating

Revisions and updating. Clearly mention the date and/or version number of the application/intervention (and comparator, if applicable) evaluated, or describe whether the intervention underwent major changes during the evaluation process, or whether the development and/or content was “frozen” during the trial. Describe dynamic components such as news feeds or changing content which may have an impact on the replicability of the intervention (for unexpected events see item 3b).

| | 1 | 2 | 3 | 4 | 5 | |
|---|---|---|---|---|---|---|
| subitem not at all important | (•) | ○ | ○ | ○ | ○ | essential |

Clear selection

Does your paper address subitem 5-iii?

Copy and paste relevant sections from the manuscript (include quotes in quotation marks "like this" to indicate direct quotes from your manuscript), or elaborate on this item by providing additional information not in the ms, or briefly explain why the item is not applicable/relevant for your study

this information is not relevant for this study

5-iv) Quality assurance methods

Provide information on quality assurance methods to ensure accuracy and quality of information provided [1], if applicable.

| | 1 | 2 | 3 | 4 | 5 | |
|---|---|---|---|---|---|---|
| subitem not at all important | (selected) | ○ | ○ | ○ | ○ | essential |

Clear selection

Does your paper address subitem 5-iv?

Copy and paste relevant sections from the manuscript (include quotes in quotation marks "like this" to indicate direct quotes from your manuscript), or elaborate on this item by providing additional information not in the ms, or briefly explain why the item is not applicable/relevant for your study

this information is not relevant for this study

5-v) Ensure replicability by publishing the source code, and/or providing screenshots/screen-capture video, and/or providing flowcharts of the algorithms used

Ensure replicability by publishing the source code, and/or providing screenshots/screen-capture video, and/or providing flowcharts of the algorithms used. Replicability (i.e., other researchers should in principle be able to replicate the study) is a hallmark of scientific reporting.

| | 1 | 2 | 3 | 4 | 5 | |
|---|---|---|---|---|---|---|
| subitem not at all important | ◉ | ○ | ○ | ○ | ○ | essential |

Clear selection

Does your paper address subitem 5-v?

Copy and paste relevant sections from the manuscript (include quotes in quotation marks "like this" to indicate direct quotes from your manuscript), or elaborate on this item by providing additional information not in the ms, or briefly explain why the item is not applicable/relevant for your study

this information is not relevant for this study

### 5-vi) Digital preservation

Digital preservation: Provide the URL of the application, but as the intervention is likely to change or disappear over the course of the years; also make sure the intervention is archived (Internet Archive, webcitation.org, and/or publishing the source code or screenshots/videos alongside the article). As pages behind login screens cannot be archived, consider creating demo pages which are accessible without login.

| | 1 | 2 | 3 | 4 | 5 | |
|---|---|---|---|---|---|---|
| subitem not at all important | (●) | ( ) | ( ) | ( ) | ( ) | essential |

Clear selection

### Does your paper address subitem 5-vi?

Copy and paste relevant sections from the manuscript (include quotes in quotation marks "like this" to indicate direct quotes from your manuscript), or elaborate on this item by providing additional information not in the ms, or briefly explain why the item is not applicable/relevant for your study

this information is not relevant for this study

5-vii) Access

Access: Describe how participants accessed the application, in what setting/context, if they had to pay (or were paid) or not, whether they had to be a member of specific group. If known, describe how participants obtained “access to the platform and Internet” [1]. To ensure access for editors/reviewers/readers, consider to provide a “backdoor” login account or demo mode for reviewers/readers to explore the application (also important for archiving purposes, see vi).

| | 1 | 2 | 3 | 4 | 5 | |
|---|---|---|---|---|---|---|
| subitem not at all important | (●) | ( ) | ( ) | ( ) | ( ) | essential |

Clear selection

Does your paper address subitem 5-vii? *

Copy and paste relevant sections from the manuscript (include quotes in quotation marks "like this" to indicate direct quotes from your manuscript), or elaborate on this item by providing additional information not in the ms, or briefly explain why the item is not applicable/relevant for your study

this information is not relevant for this study

5-viii) Mode of delivery, features/functionalities/components of the intervention and comparator, and the theoretical framework

Describe mode of delivery, features/functionalities/components of the intervention and comparator, and the theoretical framework [6] used to design them (instructional strategy [1], behaviour change techniques, persuasive features, etc., see e.g., [7, 8] for terminology). This includes an in-depth description of the content (including where it is coming from and who developed it) [1]," whether [and how] it is tailored to individual circumstances and allows users to track their progress and receive feedback" [6]. This also includes a description of communication delivery channels and – if computer-mediated communication is a component – whether communication was synchronous or asynchronous [6]. It also includes information on presentation strategies [1], including page design principles, average amount of text on pages, presence of hyperlinks to other resources, etc. [1].

| | 1 | 2 | 3 | 4 | 5 | |
|---|---|---|---|---|---|---|
| subitem not at all important | ○ | ○ | ○ | ◉ | ○ | essential |

Clear selection

Does your paper address subitem 5-viii? *

Copy and paste relevant sections from the manuscript (include quotes in quotation marks "like this" to indicate direct quotes from your manuscript), or elaborate on this item by providing additional information not in the ms, or briefly explain why the item is not applicable/relevant for your study

LARKSPUR Intervention. Individuals randomized to LARKSPUR received skills training to increase PA. The web-based intervention was self-guided and targeted eight PA skills over five weekly learning modules. The eight skills included, (1) noticing positive events [46,47]; (2) savoring positive events [48,49]; (3) identifying personal strengths [50,51]; (4) behavioral activation to set and work toward attainable goals [52,53]; (5) mindfulness [54,55]; (6) positive reappraisal [36,56]; (7) gratitude [57,58]; and (8) acts of kindness [59,60]. Each module consisted of a video introduction, interactive exercises, examples, and homework assignments. Participants practiced skills daily and reported experiences on the online platform.

Control Program. Control participants completed daily online emotion reports, rating positive and negative emotions over the past 24 hours on a 5-point Likert scale. This control condition was designed to match the LARKSPUR group in terms of online contact, attention to emotional states, and study duration, without providing any specific skills or strategies to enhance positive affect or cope with pain. Previous studies have used similar emotion reporting as a control condition for positive psychology interventions [35].

5-ix) Describe use parameters

Describe use parameters (e.g., intended "doses" and optimal timing for use). Clarify what instructions or recommendations were given to the user, e.g., regarding timing, frequency, heaviness of use, if any, or was the intervention used ad libitum.

| | 1 | 2 | 3 | 4 | 5 | |
|---|---|---|---|---|---|---|
| subitem not at all important | (•) | ( ) | ( ) | ( ) | ( ) | essential |

Clear selection

Does your paper address subitem 5-ix?

Copy and paste relevant sections from the manuscript (include quotes in quotation marks "like this" to indicate direct quotes from your manuscript), or elaborate on this item by providing additional information not in the ms, or briefly explain why the item is not applicable/relevant for your study

this information is not relevant for this study

5-x) Clarify the level of human involvement

Clarify the level of human involvement (care providers or health professionals, also technical assistance) in the e-intervention or as co-intervention (detail number and expertise of professionals involved, if any, as well as "type of assistance offered, the timing and frequency of the support, how it is initiated, and the medium by which the assistance is delivered". It may be necessary to distinguish between the level of human involvement required for the trial, and the level of human involvement required for a routine application outside of a RCT setting (discuss under item 21 – generalizability).

| | 1 | 2 | 3 | 4 | 5 | |
|---|---|---|---|---|---|---|
| subitem not at all important | ○ | ○ | ◉ | ○ | ○ | essential |

Clear selection

Does your paper address subitem 5-x?

Copy and paste relevant sections from the manuscript (include quotes in quotation marks "like this" to indicate direct quotes from your manuscript), or elaborate on this item by providing additional information not in the ms, or briefly explain why the item is not applicable/relevant for your study

The self-guided web-based intervention targeted eight PA skills over five weekly learning modules.

5-xi) Report any prompts/reminders used

Report any prompts/reminders used: Clarify if there were prompts (letters, emails, phone calls, SMS) to use the application, what triggered them, frequency etc. It may be necessary to distinguish between the level of prompts/reminders required for the trial, and the level of prompts/reminders for a routine application outside of a RCT setting (discuss under item 21 – generalizability).

| | 1 | 2 | 3 | 4 | 5 | |
|---|---|---|---|---|---|---|
| subitem not at all important | (selected) | ○ | ○ | ○ | ○ | essential |

Clear selection

Does your paper address subitem 5-xi? *

Copy and paste relevant sections from the manuscript (include quotes in quotation marks "like this" to indicate direct quotes from your manuscript), or elaborate on this item by providing additional information not in the ms, or briefly explain why the item is not applicable/relevant for your study

this information is not relevant for this study

### 5-xii) Describe any co-interventions (incl. training/support)

Describe any co-interventions (incl. training/support): Clearly state any interventions that are provided in addition to the targeted eHealth intervention, as ehealth intervention may not be designed as stand-alone intervention. This includes training sessions and support [1]. It may be necessary to distinguish between the level of training required for the trial, and the level of training for a routine application outside of a RCT setting (discuss under item 21 – generalizability.

| | 1 | 2 | 3 | 4 | 5 | |
|---|---|---|---|---|---|---|
| subitem not at all important | (•) | ( ) | ( ) | ( ) | ( ) | essential |

Clear selection

### Does your paper address subitem 5-xii? *

Copy and paste relevant sections from the manuscript (include quotes in quotation marks "like this" to indicate direct quotes from your manuscript), or elaborate on this item by providing additional information not in the ms, or briefly explain why the item is not applicable/relevant for your study

this information is not relevant for this study

### 6a) Completely defined pre-specified primary and secondary outcome measures, including how and when they were assessed

Does your paper address CONSORT subitem 6a? *

Copy and paste relevant sections from the manuscript (include quotes in quotation marks "like this" to indicate direct quotes from your manuscript), or elaborate on this item by providing additional information not in the ms, or briefly explain why the item is not applicable/relevant for your study

Positive events were assessed with 5 items asking participants to report whether the following positive events had occurred in the past 24 hours: (a) positive interpersonal interaction, (b) positive experience at work, school, or at a volunteer position, (c) positive experience at home, (d) network positive event (i.e., positive event experienced by a close friend or relative), and (e) any other positive event [10].

Positive and negative affect were assessed using the 20-item modified Differential Emotions Scale (mDES), which asked respondents to rate how often they felt 10 positive (e.g., amusement, gratitude, joy) and 10 negative (e.g., anger, guilt, sadness) emotions during the past 24 hours [61]. Items were rated on a 5-point Likert-type scale ranging from 0 ("never") to 4 ("most of the time").

Pain intensity and fatigue were each measured using a single item with a rating scale from 0 (" no pain / fatigue") to 10 ("as much pain / fatigue as could be").

6a-i) Online questionnaires: describe if they were validated for online use and apply CHERRIES items to describe how the questionnaires were designed/deployed

If outcomes were obtained through online questionnaires, describe if they were validated for online use and apply CHERRIES items to describe how the questionnaires were designed/deployed [9].

| | 1 | 2 | 3 | 4 | 5 | |
|---|---|---|---|---|---|---|
| subitem not at all important | ◉ | ○ | ○ | ○ | ○ | essential |

Clear selection

Does your paper address subitem 6a-i?

Copy and paste relevant sections from manuscript text

this information is not relevant for this study

6a-ii) Describe whether and how “use” (including intensity of use/dosage) was defined/measured/monitored

Describe whether and how “use” (including intensity of use/dosage) was defined/measured/monitored (logins, logfile analysis, etc.). Use/adoption metrics are important process outcomes that should be reported in any ehealth trial.

| | 1 | 2 | 3 | 4 | 5 | |
|---|---|---|---|---|---|---|
| subitem not at all important | (selected) | | | | | essential |

Clear selection

Does your paper address subitem 6a-ii?

Copy and paste relevant sections from manuscript text

this information is not relevant for this study

6a-iii) Describe whether, how, and when qualitative feedback from participants was obtained

Describe whether, how, and when qualitative feedback from participants was obtained (e.g., through emails, feedback forms, interviews, focus groups).

| | 1 | 2 | 3 | 4 | 5 | |
|---|---|---|---|---|---|---|
| subitem not at all important | (●) | ( ) | ( ) | ( ) | ( ) | essential |

Clear selection

Does your paper address subitem 6a-iii?

Copy and paste relevant sections from manuscript text

this information is not relevant for this study

6b) Any changes to trial outcomes after the trial commenced, with reasons

Does your paper address CONSORT subitem 6b? *

Copy and paste relevant sections from the manuscript (include quotes in quotation marks "like this" to indicate direct quotes from your manuscript), or elaborate on this item by providing additional information not in the ms, or briefly explain why the item is not applicable/relevant for your study

this information is not relevant for this study

7a) How sample size was determined

NPT: When applicable, details of whether and how the clustering by care provides or centers was addressed

7a-i) Describe whether and how expected attrition was taken into account when calculating the sample size

Describe whether and how expected attrition was taken into account when calculating the sample size.

| | 1 | 2 | 3 | 4 | 5 | |
|---|---|---|---|---|---|---|
| subitem not at all important | ○ | ○ | ○ | ◉ | ○ | essential |

Clear selection

Does your paper address subitem 7a-i?

Copy and paste relevant sections from manuscript title (include quotes in quotation marks "like this" to indicate direct quotes from your manuscript), or elaborate on this item by providing additional information not in the ms, or briefly explain why the item is not applicable/relevant for your study

Outcomes were assessed using intention-to-treat analyses for all participants providing data at baseline and at least one post-intervention or follow-up assessment. Data were modeled using multivariate longitudinal mixed-effects models [72] within a Bayesian framework [73] using the brms software package (Version 2.20.4) [74] with default noninformative priors. Specifically, we modeled PA and NA as bivariate outcomes where PA was normally distributed and NA was lognormally distributed to account for notable positive skew potentially arising from censoring [75] (regression coefficients for NA represent multiplicative change in NA for a one-unit predictor increase); we modeled pain and fatigue as correlated multivariate normally distributed outcomes.

7b) When applicable, explanation of any interim analyses and stopping guidelines

Does your paper address CONSORT subitem 7b? *

Copy and paste relevant sections from the manuscript (include quotes in quotation marks "like this" to indicate direct quotes from your manuscript), or elaborate on this item by providing additional information not in the ms, or briefly explain why the item is not applicable/relevant for your study

this information is not relevant for this study

8a) Method used to generate the random allocation sequence

NPT: When applicable, how care providers were allocated to each trial group

Does your paper address CONSORT subitem 8a? *

Copy and paste relevant sections from the manuscript (include quotes in quotation marks "like this" to indicate direct quotes from your manuscript), or elaborate on this item by providing additional information not in the ms, or briefly explain why the item is not applicable/relevant for your study

this information is not relevant for this study

8b) Type of randomisation; details of any restriction (such as blocking and block size)

Does your paper address CONSORT subitem 8b? *

Copy and paste relevant sections from the manuscript (include quotes in quotation marks "like this" to indicate direct quotes from your manuscript), or elaborate on this item by providing additional information not in the ms, or briefly explain why the item is not applicable/relevant for your study

this information is not relevant for this study

9) Mechanism used to implement the random allocation sequence (such as sequentially numbered containers), describing any steps taken to conceal the sequence until interventions were assigned

Does your paper address CONSORT subitem 9? *

Copy and paste relevant sections from the manuscript (include quotes in quotation marks "like this" to indicate direct quotes from your manuscript), or elaborate on this item by providing additional information not in the ms, or briefly explain why the item is not applicable/relevant for your study

this information is not relevant for this study

10) Who generated the random allocation sequence, who enrolled participants, and who assigned participants to interventions

Does your paper address CONSORT subitem 10? *

Copy and paste relevant sections from the manuscript (include quotes in quotation marks "like this" to indicate direct quotes from your manuscript), or elaborate on this item by providing additional information not in the ms, or briefly explain why the item is not applicable/relevant for your study

this information is not relevant for this study

11a) If done, who was blinded after assignment to interventions (for example, participants, care providers, those assessing outcomes) and how

NPT: Whether or not administering co-interventions were blinded to group assignment

11a-i) Specify who was blinded, and who wasn't

Specify who was blinded, and who wasn't. Usually, in web-based trials it is not possible to blind the participants [1, 3] (this should be clearly acknowledged), but it may be possible to blind outcome assessors, those doing data analysis or those administering co-interventions (if any).

| | 1 | 2 | 3 | 4 | 5 | |
|---|---|---|---|---|---|---|
| subitem not at all important | ◉ | ○ | ○ | ○ | ○ | essential |

Clear selection

Does your paper address subitem 11a-i? *

Copy and paste relevant sections from the manuscript (include quotes in quotation marks "like this" to indicate direct quotes from your manuscript), or elaborate on this item by providing additional information not in the ms, or briefly explain why the item is not applicable/relevant for your study

this information is not relevant for this study

11a-ii) Discuss e.g., whether participants knew which intervention was the "intervention of interest" and which one was the "comparator"

Informed consent procedures (4a-ii) can create biases and certain expectations - discuss e.g., whether participants knew which intervention was the "intervention of interest" and which one was the "comparator".

| | 1 | 2 | 3 | 4 | 5 | |
|---|---|---|---|---|---|---|
| subitem not at all important | ◉ | ○ | ○ | ○ | ○ | essential |

Clear selection

Does your paper address subitem 11a-ii?

Copy and paste relevant sections from the manuscript (include quotes in quotation marks "like this" to indicate direct quotes from your manuscript), or elaborate on this item by providing additional information not in the ms, or briefly explain why the item is not applicable/relevant for your study

this information is not relevant for this study

11b) If relevant, description of the similarity of interventions

(this item is usually not relevant for ehealth trials as it refers to similarity of a placebo or sham intervention to a active medication/intervention)

Does your paper address CONSORT subitem 11b? *

Copy and paste relevant sections from the manuscript (include quotes in quotation marks "like this" to indicate direct quotes from your manuscript), or elaborate on this item by providing additional information not in the ms, or briefly explain why the item is not applicable/relevant for your study

this information is not relevant for this study

12a) Statistical methods used to compare groups for primary and secondary outcomes

NPT: When applicable, details of whether and how the clustering by care providers or centers was addressed

Does your paper address CONSORT subitem 12a? *

Copy and paste relevant sections from the manuscript (include quotes in quotation marks "like this" to indicate direct quotes from your manuscript), or elaborate on this item by providing additional information not in the ms, or briefly explain why the item is not applicable/relevant for your study

Given the relatively modest sample size and longitudinal data structure, we utilized Bayesian estimation rather than frequentist estimation. Compared to frequentism, Bayesian methods are better powered for modeling complex data with limited observations, as they incorporate both sample evidence and prior knowledge to derive posterior distributions [62]. To enable effect size interpretation, we report Bayesian analogs to frequentist intervals and p-values: highest posterior density intervals (HPDs) and posterior probabilities of direction (pd). HPDs describe uncertainty by delineating the most credible values comprising a certain percentage of the posterior distribution [63]. For example, a 95% HPD of 0.1 to 0.3 indicates a 95% probability that the true effect lies between 0.1 and 0.3 based on the accumulated evidence. The pd value directly quantifies certainty regarding an effect's existence and direction [64].

We computed pd using the bayestestR software (Version 0.13.1) [65]. Though related to frequentist p-values, pd offers advantages in interpreting effects for small samples [66]. If 99% of the posterior distribution lies above zero, there is high certainty of a positive effect. We used a pd threshold of 0.975, corresponding to a frequentist significance level of 0.05 for two-sided testing. Overall, this Bayesian framework enabled well-powered hypothesis testing despite the limited sample size.

12a-i) Imputation techniques to deal with attrition / missing values

Imputation techniques to deal with attrition / missing values: Not all participants will use the intervention/comparator as intended and attrition is typically high in ehealth trials. Specify how participants who did not use the application or dropped out from the trial were treated in the statistical analysis (a complete case analysis is strongly discouraged, and simple imputation techniques such as LOCF may also be problematic [4]).

| | 1 | 2 | 3 | 4 | 5 | |
|---|---|---|---|---|---|---|
| subitem not at all important | ○ | ○ | ○ | ◉ | ○ | essential |

Clear selection

Does your paper address subitem 12a-i? *

Copy and paste relevant sections from the manuscript (include quotes in quotation marks "like this" to indicate direct quotes from your manuscript), or elaborate on this item by providing additional information not in the ms, or briefly explain why the item is not applicable/relevant for your study

Outcomes were assessed using intention-to-treat analyses for all participants providing data at baseline and at least one post-intervention or follow-up assessment. Data were modeled using multivariate longitudinal mixed-effects models [72] within a Bayesian framework [73] using the brms software package (Version 2.20.4) [74] with default noninformative priors. Specifically, we modeled PA and NA as bivariate outcomes where PA was normally distributed and NA was lognormally distributed to account for notable positive skew potentially arising from censoring [75] (regression coefficients for NA represent multiplicative change in NA for a one-unit predictor increase); we modeled pain and fatigue as correlated multivariate normally distributed outcomes.

12b) Methods for additional analyses, such as subgroup analyses and adjusted analyses

Does your paper address CONSORT subitem 12b? *

Copy and paste relevant sections from the manuscript (include quotes in quotation marks "like this" to indicate direct quotes from your manuscript), or elaborate on this item by providing additional information not in the ms, or briefly explain why the item is not applicable/relevant for your study

Between-person and within-person effects of PE were disaggregated by including individual burst-specific average PE (grand-mean-centered) and person-mean-centered daily PE as predictors of all outcomes [76]. Finally, we assessed moderation effects of within-person and between-person PE on treatment differences and treatment differences in change by including three-way interactions (Average PE x Group x Time; Daily PE x Group x Time) and lower-order two-way interactions among treatment group, time, and daily/average PE. Planned comparisons examined between-group differences in PE responsivity at post-treatment and 1-month follow-up.

X26) REB/IRB Approval and Ethical Considerations [recommended as subheading under "Methods"] (not a CONSORT item)

X26-i) Comment on ethics committee approval

| subitem not at all important | 1 | 2 | 3 | 4 | 5 | essential |
|---|---|---|---|---|---|---|
| | ○ | ○ | ○ | ◉ | ○ | |

Clear selection

Does your paper address subitem X26-i?

Copy and paste relevant sections from the manuscript (include quotes in quotation marks "like this" to indicate direct quotes from your manuscript), or elaborate on this item by providing additional information not in the ms, or briefly explain why the item is not applicable/relevant for your study

The study was reviewed and approved by the Institutional Review Board (IRB) at Weill Cornell Medicine

x26-ii) Outline informed consent procedures

Outline informed consent procedures e.g., if consent was obtained offline or online (how? Checkbox, etc.?), and what information was provided (see 4a-ii). See [6] for some items to be included in informed consent documents.

| subitem not at all important | 1 | 2 | 3 | 4 | 5 | essential |
|---|---|---|---|---|---|---|
| | ○ | ○ | ◉ | ○ | ○ | |

Clear selection

Does your paper address subitem X26-ii?

Copy and paste relevant sections from the manuscript (include quotes in quotation marks "like this" to indicate direct quotes from your manuscript), or elaborate on this item by providing additional information not in the ms, or briefly explain why the item is not applicable/relevant for your study

Eligible and consenting participants were randomized to LARKSPUR or control.

X26-iii) Safety and security procedures

Safety and security procedures, incl. privacy considerations, and any steps taken to reduce the likelihood or detection of harm (e.g., education and training, availability of a hotline)

| | 1 | 2 | 3 | 4 | 5 | |
|---|---|---|---|---|---|---|
| subitem not at all important | ○ | ◉ | ○ | ○ | ○ | essential |

Clear selection

Does your paper address subitem X26-iii?

Copy and paste relevant sections from the manuscript (include quotes in quotation marks "like this" to indicate direct quotes from your manuscript), or elaborate on this item by providing additional information not in the ms, or briefly explain why the item is not applicable/relevant for your study

this information is not relevant for this study

RESULTS

13a) For each group, the numbers of participants who were randomly assigned, received intended treatment, and were analysed for the primary outcome

NPT: The number of care providers or centers performing the intervention in each group and the number of patients treated by each care provider in each center

Does your paper address CONSORT subitem 13a? *

Copy and paste relevant sections from the manuscript (include quotes in quotation marks "like this" to indicate direct quotes from your manuscript), or elaborate on this item by providing additional information not in the ms, or briefly explain why the item is not applicable/relevant for your study

Outcomes were assessed using intention-to-treat analyses for all participants providing data at baseline and at least one post-intervention or follow-up assessment. Data were modeled using multivariate longitudinal mixed-effects models [72] within a Bayesian framework [73] using the brms software package (Version 2.20.4) [74] with default noninformative priors. Specifically, we modeled PA and NA as bivariate outcomes where PA was normally distributed and NA was lognormally distributed to account for notable positive skew potentially arising from censoring [75] (regression coefficients for NA represent multiplicative change in NA for a one-unit predictor increase); we modeled pain and fatigue as correlated multivariate normally distributed outcomes.

13b) For each group, losses and exclusions after randomisation, together with reasons

Does your paper address CONSORT subitem 13b? (NOTE: Preferably, this is shown in a CONSORT flow diagram) *

Copy and paste relevant sections from the manuscript (include quotes in quotation marks "like this" to indicate direct quotes from your manuscript), or elaborate on this item by providing additional information not in the ms, or briefly explain why the item is not applicable/relevant for your study

Of 142 individuals screened for participation, 95 were eligible and enrolled in the study. All 95 participants completed the baseline questionnaires and were randomized to the LARKSPUR intervention (n = 49) or the control (n = 46). Using an intent-to-treat analysis, 86 participants (n = 43 LARKSPUR, n = 43 control) completing baseline and post-intervention assessments were included. In addition, participants completing ≥ 2 daily assessments per measurement burst were included to assess daily changes, resulting in a final analytic sample of 80 participants (n = 40 in LARKSPUR, n = 40 in control).

13b-i) Attrition diagram

Strongly recommended: An attrition diagram (e.g., proportion of participants still logging in or using the intervention/comparator in each group plotted over time, similar to a survival curve) or other figures or tables demonstrating usage/dose/engagement.

| | 1 | 2 | 3 | 4 | 5 | |
|---|---|---|---|---|---|---|
| subitem not at all important | (●) | ( ) | ( ) | ( ) | ( ) | essential |

Clear selection

Does your paper address subitem 13b-i?

Copy and paste relevant sections from the manuscript or cite the figure number if applicable (include quotes in quotation marks "like this" to indicate direct quotes from your manuscript), or elaborate on this item by providing additional information not in the ms, or briefly explain why the item is not applicable/relevant for your study

this information is not relevant for this study

14a) Dates defining the periods of recruitment and follow-up

Does your paper address CONSORT subitem 14a? *

Copy and paste relevant sections from the manuscript (include quotes in quotation marks "like this" to indicate direct quotes from your manuscript), or elaborate on this item by providing additional information not in the ms, or briefly explain why the item is not applicable/relevant for your study

Participants in both arms were assessed at baseline, at 8 weeks (post-intervention), and 1-month follow-up. In addition, before (baseline) and after the intervention (post) and at 1-month follow-up, participants completed a 7-day burst of online daily assessments of positive events, positive affect (PA) and negative affect (NA), pain intensity, and fatigue.

14a-i) Indicate if critical "secular events" fell into the study period

Indicate if critical "secular events" fell into the study period, e.g., significant changes in Internet resources available or "changes in computer hardware or Internet delivery resources"

| | 1 | 2 | 3 | 4 | 5 | |
|---|---|---|---|---|---|---|
| subitem not at all important | (selected) | ○ | ○ | ○ | ○ | essential |

Clear selection

Does your paper address subitem 14a-i?

Copy and paste relevant sections from the manuscript (include quotes in quotation marks "like this" to indicate direct quotes from your manuscript), or elaborate on this item by providing additional information not in the ms, or briefly explain why the item is not applicable/relevant for your study

this information is not relevant for this study

14b) Why the trial ended or was stopped (early)

Does your paper address CONSORT subitem 14b? *

Copy and paste relevant sections from the manuscript (include quotes in quotation marks "like this" to indicate direct quotes from your manuscript), or elaborate on this item by providing additional information not in the ms, or briefly explain why the item is not applicable/relevant for your study

this information is not relevant for this study

15) A table showing baseline demographic and clinical characteristics for each group

NPT: When applicable, a description of care providers (case volume, qualification, expertise, etc.) and centers (volume) in each group

Does your paper address CONSORT subitem 15? *

Copy and paste relevant sections from the manuscript (include quotes in quotation marks "like this" to indicate direct quotes from your manuscript), or elaborate on this item by providing additional information not in the ms, or briefly explain why the item is not applicable/relevant for your study

Table 1. Demographic characteristics and baseline measures of participants in LARKSPUR (N = 40) and Control (N = 40)

Variable LARKSPUR
N (%) Control
N (%) Overall
N (%)
Gender
Male
Female
1 (2.5)
39 (97.5)
3 (7.5)
37 (92.5)
4 (5)
76 (95)
Age (years)
50—59
60—69
70—79
≥ 80
17 (42.5)
16 (40)
6 (15)
1 (2.5)
21 (52.5)
17 (42.5)
2 (5)
0 (0)
38 (47.5)
33 (41.25)
8 (10)
1 (1.25)
Ethnicity
Hispanic or Latino
Black or African American
White
More than one race
Not Reported
1 (2.5)
2 (5)
33 (82.5)
3 (7.5)
1 (2.5)
2 (5)
4 (10)
32 (80)
1 (2.5)
1 (2.5)
[illegible]

3 (3.75)
6 (7.5)
65 (81.25)
4 (5)
3 (3.75)
Education
High School Diploma or GED
Some College (No Degree)
Associate Degree
Bachelor’s Degree
Post-graduate (No Degree)
Master’s Degree
Doctoral Degree
2 (5)
9 (22.5)
6 (15)
10 (25)
3 (7.5)
8 (20)
2 (5)
1 (2.5)
8 (20)
4 (10)
8 (20)
7 (17.5)
12 (30)
0 (0)
3 (3)
17 (20)
11 (13)
21 (24)
10 (12)
21 (24)
3 (3)

| Baseline Measure | M (SD) | M (SD) | M (SD) |
|---|---|---|---|
| Positive Events | 2.2 (0.9) | 2.3 (1.0) | 2.2 (0.9) |
| Positive Affect | 2.7 (0.6) | 2.7 (0.6) | 2.7 (0.6) |
| Negative Affect | 1.7 (0.5) | 1.5 (0.5) | 1.6 (0.5) |
| Pain | 5.5 (1.5) | 5.5 (1.8) | 5.5 (1.7) |
| Fatigue | 6.0 (1.7) | 5.4 (2.1) | 5.7 (1.9) |

Note. N = total within column, % = percentage of total within column, M = mean, SD = standard deviation.

15-i) Report demographics associated with digital divide issues

In ehealth trials it is particularly important to report demographics associated with digital divide issues, such as age, education, gender, social-economic status, computer/Internet/ehealth literacy of the participants, if known.

| | 1 | 2 | 3 | 4 | 5 | |
|---|---|---|---|---|---|---|
| subitem not at all important | ◉ | ○ | ○ | ○ | ○ | essential |

Clear selection

Does your paper address subitem 15-i? *

Copy and paste relevant sections from the manuscript (include quotes in quotation marks "like this" to indicate direct quotes from your manuscript), or elaborate on this item by providing additional information not in the ms, or briefly explain why the item is not applicable/relevant for your study

this information is not relevant for this study

16) For each group, number of participants (denominator) included in each analysis and whether the analysis was by original assigned groups

16-i) Report multiple “denominators” and provide definitions

Report multiple “denominators” and provide definitions: Report N’s (and effect sizes) “across a range of study participation [and use] thresholds” [1], e.g., N exposed, N consented, N used more than x times, N used more than y weeks, N participants “used” the intervention/comparator at specific pre-defined time points of interest (in absolute and relative numbers per group). Always clearly define “use” of the intervention.

1 2 3 4 5

(•) 1 ( ) 2 ( ) 3 ( ) 4 ( ) 5

subitem not at all important essential

Clear selection

Does your paper address subitem 16-i? *

Copy and paste relevant sections from the manuscript (include quotes in quotation marks "like this" to indicate direct quotes from your manuscript), or elaborate on this item by providing additional information not in the ms, or briefly explain why the item is not applicable/relevant for your study

this information is not relevant for this study

16-ii) Primary analysis should be intent-to-treat

Primary analysis should be intent-to-treat, secondary analyses could include comparing only “users”, with the appropriate caveats that this is no longer a randomized sample (see 18-i).

1 2 3 4 5

( ) 1 ( ) 2 (•) 3 ( ) 4 ( ) 5

subitem not at all important essential

Clear selection

Does your paper address subitem 16-ii?

Copy and paste relevant sections from the manuscript (include quotes in quotation marks "like this" to indicate direct quotes from your manuscript), or elaborate on this item by providing additional information not in the ms, or briefly explain why the item is not applicable/relevant for your study

Using an intent-to-treat analysis, 86 participants (n = 43 LARKSPUR, n = 43 control) completing baseline and post-intervention assessments were included. In addition, participants completing ≥ 2 daily assessments per measurement burst were included to assess daily changes, resulting in a final analytic sample of 80 participants (n = 40 in LARKSPUR, n = 40 in control).

17a) For each primary and secondary outcome, results for each group, and the estimated effect size and its precision (such as 95% confidence interval)

Does your paper address CONSORT subitem 17a? *

Copy and paste relevant sections from the manuscript (include quotes in quotation marks "like this" to indicate direct quotes from your manuscript), or elaborate on this item by providing additional information not in the ms, or briefly explain why the item is not applicable/relevant for your study

Affective responsivity. The bivariate mixed-effects model revealed significant interactions between treatment group, time point, and daily PE in predicting PA and NA. Parameter estimates and 95% credible intervals are presented in Table 2. These interactions indicate that LARKSPUR differentially impacted affective responsivity to daily PE compared to the control condition. Specifically, planned comparisons showed that at post-treatment, LARKSPUR participants exhibited greater reductions in NA (bL – bC = -0.06, 95% BCI: [-0.10, -0.02], pd = .997) and increases in PA (bL – bC = 0.10, 95% BCI: [0.02, 0.19], pd = .991) in response to daily PE compared to controls. However, these differential gains in affective responsivity were not maintained at 1-month follow-up, with non-significant between-group differences in both PA (bL – bC = 0.01, 95% BCI: [-0.08, 0.09], pd = .555) and NA (bL – bC = -0.00, 95% BCI: [-0.04, 0.04], pd = .513) responsivity. As shown in Figure 1, both groups evidenced slightly diminished affective responsivity from post-treatment to follow-up, suggesting that continued practice of LARKSPUR skills may be necessary to sustain affective gains long-term.

Pain and fatigue responsivity. In contrast to the affective outcomes, LARKSPUR yielded more persistent improvements in daily pain and fatigue responsivity compared to control (see Table 2). Across posttreatment and follow-up, LARKSPUR led to greater reductions in pain (bL – bC = -0.20, 95% BCI: [-0.36, -0.04], pd = .994) and fatigue (bL – bC = -0.24, 95% BCI: [-0.41, -0.06], pd = .996) following positive events. As illustrated in Figure 2, LARKSPUR participants maintained diminished pain and fatigue following daily PE from post-treatment through follow-up, whereas controls showed minimal change. This sustained effect for the functional outcomes indicates that LARKSPUR may have produced broader impacts beyond temporary affective gains.

17a-i) Presentation of process outcomes such as metrics of use and intensity of use

In addition to primary/secondary (clinical) outcomes, the presentation of process outcomes such as metrics of use and intensity of use (dose, exposure) and their operational definitions is critical. This does not only refer to metrics of attrition (13-b) (often a binary variable), but also to more continuous exposure metrics such as “average session length”. These must be accompanied by a technical description how a metric like a “session” is defined (e.g., timeout after idle time) [1] (report under item 6a).

| | 1 | 2 | 3 | 4 | 5 | |
|---|---|---|---|---|---|---|
| subitem not at all important | ◉ | ○ | ○ | ○ | ○ | essential |

Clear selection

Does your paper address subitem 17a-i?

Copy and paste relevant sections from the manuscript (include quotes in quotation marks "like this" to indicate direct quotes from your manuscript), or elaborate on this item by providing additional information not in the ms, or briefly explain why the item is not applicable/relevant for your study

this information is not relevant for this study

17b) For binary outcomes, presentation of both absolute and relative effect sizes is recommended

Does your paper address CONSORT subitem 17b? *

Copy and paste relevant sections from the manuscript (include quotes in quotation marks "like this" to indicate direct quotes from your manuscript), or elaborate on this item by providing additional information not in the ms, or briefly explain why the item is not applicable/relevant for your study

this information is not relevant for this study

18) Results of any other analyses performed, including subgroup analyses and adjusted analyses, distinguishing pre-specified from exploratory

Does your paper address CONSORT subitem 18? *

Copy and paste relevant sections from the manuscript (include quotes in quotation marks "like this" to indicate direct quotes from your manuscript), or elaborate on this item by providing additional information not in the ms, or briefly explain why the item is not applicable/relevant for your study

Affective responsivity. The bivariate mixed-effects model revealed significant interactions between treatment group, time point, and daily PE in predicting PA and NA. Parameter estimates and 95% credible intervals are presented in Table 2. These interactions indicate that LARKSPUR differentially impacted affective responsivity to daily PE compared to the control condition. Specifically, planned comparisons showed that at post-treatment, LARKSPUR participants exhibited greater reductions in NA (bL – bC = -0.06, 95% BCI: [-0.10, -0.02], pd = .997) and increases in PA (bL – bC = 0.10, 95% BCI: [0.02, 0.19], pd = .991) in response to daily PE compared to controls. However, these differential gains in affective responsivity were not maintained at 1-month follow-up, with non-significant between-group differences in both PA (bL – bC = 0.01, 95% BCI: [-0.08, 0.09], pd = .555) and NA (bL – bC = -0.00, 95% BCI: [-0.04, 0.04], pd = .513) responsivity. As shown in Figure 1, both groups evidenced slightly diminished affective responsivity from post-treatment to follow-up, suggesting that continued practice of LARKSPUR skills may be necessary to sustain affective gains long-term.

Pain and fatigue responsivity. In contrast to the affective outcomes, LARKSPUR yielded more persistent improvements in daily pain and fatigue responsivity compared to control (see Table 2). Across posttreatment and follow-up, LARKSPUR led to greater reductions in pain (bL – bC = -0.20, 95% BCI: [-0.36, -0.04], pd = .994) and fatigue (bL – bC = -0.24, 95% BCI: [-0.41, -0.06], pd = .996) following positive events. As illustrated in Figure 2, LARKSPUR participants maintained diminished pain and fatigue following daily PE from post-treatment through follow-up, whereas controls showed minimal change. This sustained effect for the functional outcomes indicates that LARKSPUR may have produced broader impacts beyond temporary affective gains.

18-i) Subgroup analysis of comparing only users

A subgroup analysis of comparing only users is not uncommon in ehealth trials, but if done, it must be stressed that this is a self-selected sample and no longer an unbiased sample from a randomized trial (see 16-iii).

| | 1 | 2 | 3 | 4 | 5 | |
|---|---|---|---|---|---|---|
| subitem not at all important | ◉ | ○ | ○ | ○ | ○ | essential |

Clear selection

Does your paper address subitem 18-i?

Copy and paste relevant sections from the manuscript (include quotes in quotation marks "like this" to indicate direct quotes from your manuscript), or elaborate on this item by providing additional information not in the ms, or briefly explain why the item is not applicable/relevant for your study

this information is not relevant in this study

19) All important harms or unintended effects in each group
(for specific guidance see CONSORT for harms)

Does your paper address CONSORT subitem 19? *

Copy and paste relevant sections from the manuscript (include quotes in quotation marks "like this" to indicate direct quotes from your manuscript), or elaborate on this item by providing additional information not in the ms, or briefly explain why the item is not applicable/relevant for your study

this information is not relevant in this study

19-i) Include privacy breaches, technical problems

Include privacy breaches, technical problems. This does not only include physical “harm” to participants, but also incidents such as perceived or real privacy breaches [1], technical problems, and other unexpected/unintended incidents. “Unintended effects” also includes unintended positive effects [2].

| | 1 | 2 | 3 | 4 | 5 | |
|---|---|---|---|---|---|---|
| subitem not at all important | (•) | ( ) | ( ) | ( ) | ( ) | essential |

Clear selection

Does your paper address subitem 19-i?

Copy and paste relevant sections from the manuscript (include quotes in quotation marks "like this" to indicate direct quotes from your manuscript), or elaborate on this item by providing additional information not in the ms, or briefly explain why the item is not applicable/relevant for your study

this information is not relevant in this study

19-ii) Include qualitative feedback from participants or observations from staff/researchers

Include qualitative feedback from participants or observations from staff/researchers, if available, on strengths and shortcomings of the application, especially if they point to unintended/unexpected effects or uses. This includes (if available) reasons for why people did or did not use the application as intended by the developers.

| | 1 | 2 | 3 | 4 | 5 | |
|---|---|---|---|---|---|---|
| subitem not at all important | (•) | ( ) | ( ) | ( ) | ( ) | essential |

Clear selection

Does your paper address subitem 19-ii?

Copy and paste relevant sections from the manuscript (include quotes in quotation marks "like this" to indicate direct quotes from your manuscript), or elaborate on this item by providing additional information not in the ms, or briefly explain why the item is not applicable/relevant for your study

this information is not relevant in this study

DISCUSSION

22) Interpretation consistent with results, balancing benefits and harms, and considering other relevant evidence

NPT: In addition, take into account the choice of the comparator, lack of or partial blinding, and unequal expertise of care providers or centers in each group

22-i) Restate study questions and summarize the answers suggested by the data, starting with primary outcomes and process outcomes (use)

Restate study questions and summarize the answers suggested by the data, starting with primary outcomes and process outcomes (use).

| | 1 | 2 | 3 | 4 | 5 | |
|---|---|---|---|---|---|---|
| subitem not at all important | ○ | ○ | ○ | ◉ | ○ | essential |

Clear selection

Does your paper address subitem 22-i? *

Copy and paste relevant sections from the manuscript (include quotes in quotation marks "like this" to indicate direct quotes from your manuscript), or elaborate on this item by providing additional information not in the ms, or briefly explain why the item is not applicable/relevant for your study

This randomized controlled trial provides initial evidence that LARKSPUR, an internet-delivered positive affect skills intervention, can enhance responsivity to daily PE in adults with FMS. LARKSPUR led to greater decreases in NA and increases in PA following positive events compared to control. It also reduced led to reductions in pain and fatigue following positive events at post-treatment and at one month follow-up. These findings suggest that LARKSPUR has the potential to be a feasible, accessible, and effective eHealth intervention to boost well-being and improve symptom management in this population.

22-ii) Highlight unanswered new questions, suggest future research

Highlight unanswered new questions, suggest future research.

| | 1 | 2 | 3 | 4 | 5 | |
|---|---|---|---|---|---|---|
| subitem not at all important | ○ | ○ | ◉ | ○ | ○ | essential |

Clear selection

Does your paper address subitem 22-ii?

Copy and paste relevant sections from the manuscript (include quotes in quotation marks "like this" to indicate direct quotes from your manuscript), or elaborate on this item by providing additional information not in the ms, or briefly explain why the item is not applicable/relevant for your study

Some limitations should be noted. The modest sample size reduces precision and generalizability. Recruiting larger, more diverse fibromyalgia samples would allow examination of LARKSPUR's effects across different demographic groups. Additionally, self-report measures may bias results. Incorporating objective assessments like clinician observations or actigraphy for sleep and activity would provide convergent evidence. This study also focused solely on positive event frequency. Investigating variety in daily experiences may further illuminate paths to improving overall well-being, as exposure to diverse situations can build flexible coping skills [83–85]. Moreover, longer-term follow-up would offer insight into the sustainability of benefits and strategies such as periodic booster sessions to maintain gains

20) Trial limitations, addressing sources of potential bias, imprecision, and, if relevant, multiplicity of analyses

20-i) Typical limitations in ehealth trials

Typical limitations in ehealth trials: Participants in ehealth trials are rarely blinded. Ehealth trials often look at a multiplicity of outcomes, increasing risk for a Type I error. Discuss biases due to non-use of the intervention/usability issues, biases through informed consent procedures, unexpected events.

| | 1 | 2 | 3 | 4 | 5 | |
|---|---|---|---|---|---|---|
| subitem not at all important | ○ | ○ | ● | ○ | ○ | essential |

Clear selection

Does your paper address subitem 20-i? *

Copy and paste relevant sections from the manuscript (include quotes in quotation marks "like this" to indicate direct quotes from your manuscript), or elaborate on this item by providing additional information not in the ms, or briefly explain why the item is not applicable/relevant for your study

Some limitations should be noted. The modest sample size reduces precision and generalizability. Recruiting larger, more diverse fibromyalgia samples would allow examination of LARKSPUR's effects across different demographic groups. Additionally, self-report measures may bias results. Incorporating objective assessments like clinician observations or actigraphy for sleep and activity would provide convergent evidence. This study also focused solely on positive event frequency. Investigating variety in daily experiences may further illuminate paths to improving overall well-being, as exposure to diverse situations can build flexible coping skills [83–85]. Moreover, longer-term follow-up would offer insight into the sustainability of benefits and strategies such as periodic booster sessions to maintain gains

21) Generalisability (external validity, applicability) of the trial findings

NPT: External validity of the trial findings according to the intervention, comparators, patients, and care providers or centers involved in the trial

21-i) Generalizability to other populations

Generalizability to other populations: In particular, discuss generalizability to a general Internet population, outside of a RCT setting, and general patient population, including applicability of the study results for other organizations

| | 1 | 2 | 3 | 4 | 5 | |
|---|---|---|---|---|---|---|
| subitem not at all important | ○ | ○ | ● | ○ | ○ | essential |

Clear selection

Does your paper address subitem 21-i?

Copy and paste relevant sections from the manuscript (include quotes in quotation marks "like this" to indicate direct quotes from your manuscript), or elaborate on this item by providing additional information not in the ms, or briefly explain why the item is not applicable/relevant for your study

Some limitations should be noted. The modest sample size reduces precision and generalizability. Recruiting larger, more diverse fibromyalgia samples would allow examination of LARKSPUR's effects across different demographic groups. Additionally, self-report measures may bias results. Incorporating objective assessments like clinician observations or actigraphy for sleep and activity would provide convergent evidence. This study also focused solely on positive event frequency. Investigating variety in daily experiences may further illuminate paths to improving overall well-being, as exposure to diverse situations can build flexible coping skills [83–85]. Moreover, longer-term follow-up would offer insight into the sustainability of benefits and strategies such as periodic booster sessions to maintain gains

21-ii) Discuss if there were elements in the RCT that would be different in a routine application setting

Discuss if there were elements in the RCT that would be different in a routine application setting (e.g., prompts/reminders, more human involvement, training sessions or other co-interventions) and what impact the omission of these elements could have on use, adoption, or outcomes if the intervention is applied outside of a RCT setting.

| | 1 | 2 | 3 | 4 | 5 | |
|---|---|---|---|---|---|---|
| subitem not at all important | (selected) | ○ | ○ | ○ | ○ | essential |

Clear selection

Does your paper address subitem 21-ii?

Copy and paste relevant sections from the manuscript (include quotes in quotation marks "like this" to indicate direct quotes from your manuscript), or elaborate on this item by providing additional information not in the ms, or briefly explain why the item is not applicable/relevant for your study

this information is not relevant in this study

OTHER INFORMATION

23) Registration number and name of trial registry

Does your paper address CONSORT subitem 23? *

Copy and paste relevant sections from the manuscript (include quotes in quotation marks "like this" to indicate direct quotes from your manuscript), or elaborate on this item by providing additional information not in the ms, or briefly explain why the item is not applicable/relevant for your study

Trial Registration: NCT04869345

24) Where the full trial protocol can be accessed, if available

Does your paper address CONSORT subitem 24? *

Cite a Multimedia Appendix, other reference, or copy and paste relevant sections from the manuscript (include quotes in quotation marks "like this" to indicate direct quotes from your manuscript), or elaborate on this item by providing additional information not in the ms, or briefly explain why the item is not applicable/relevant for your study

this information is not relevant for this paper

25) Sources of funding and other support (such as supply of drugs), role of funders

Does your paper address CONSORT subitem 25? *

Copy and paste relevant sections from the manuscript (include quotes in quotation marks "like this" to indicate direct quotes from your manuscript), or elaborate on this item by providing additional information not in the ms, or briefly explain why the item is not applicable/relevant for your study

This work was supported by the National Institute on Aging of the National Institutes of Health (grant number R24AG064191).

X27) Conflicts of Interest (not a CONSORT item)

X27-i) State the relation of the study team towards the system being evaluated

In addition to the usual declaration of interests (financial or otherwise), also state the relation of the study team towards the system being evaluated, i.e., state if the authors/evaluators are distinct from or identical with the developers/sponsors of the intervention.

| | 1 | 2 | 3 | 4 | 5 | |
|---|---|---|---|---|---|---|
| subitem not at all important | ○ | ○ | ○ | ◉ | ○ | essential |

Clear selection

Does your paper address subitem X27-i?

Copy and paste relevant sections from the manuscript (include quotes in quotation marks "like this" to indicate direct quotes from your manuscript), or elaborate on this item by providing additional information not in the ms, or briefly explain why the item is not applicable/relevant for your study

No conflict of interest in this study

## About the CONSORT EHEALTH checklist

As a result of using this checklist, did you make changes in your manuscript? *

- [ ] yes, major changes
- [x] yes, minor changes
- [ ] no

What were the most important changes you made as a result of using this checklist?

terminology with respect to mode of delivery

How much time did you spend on going through the checklist INCLUDING making changes in your manuscript *

1 hour going through this checklist

As a result of using this checklist, do you think your manuscript has improved? *

- (x) yes
- ( ) no
- ( ) Other:

Would you like to become involved in the CONSORT EHEALTH group?

This would involve for example becoming involved in participating in a workshop and writing an "Explanation and Elaboration" document

- ( ) yes
- (x) no
- ( ) Other:

Clear selection

Any other comments or questions on CONSORT EHEALTH

Your answer

STOP - Save this form as PDF before you click submit

To generate a record that you filled in this form, we recommend to generate a PDF of this page (on a Mac, simply select "print" and then select "print as PDF") before you submit it.

When you submit your (revised) paper to JMIR, please upload the PDF as supplementary file.

Don't worry if some text in the textboxes is cut off, as we still have the complete information in our database. Thank you!

Final step: Click submit !

Click submit so we have your answers in our database!

Submit Clear form

Never submit passwords through Google Forms.

This content is neither created nor endorsed by Google. Report Abuse - Terms of Service - Privacy Policy

Google Forms
